# Psychosocial burden of axial spondyloarthritis and impact of different disease domains: a systematic literature review

**DOI:** 10.1093/rap/rkaf063

**Published:** 2025-05-26

**Authors:** Denis Poddubnyy, Uta Kiltz, Abhijeet Danve, Grace Wright, Rebecca Haberman, Ana Biljan, Jerry Clewell, Jamie Urbanik, Heather Jones, Marina Magrey

**Affiliations:** Division of Rheumatology, Department of Medicine, University of Toronto, Toronto, Canada; Department of Gastroenterology, Infectious Diseases and Rheumatology, Charité-Universitätsmedizin Berlin, Berlin, Germany; Rheumatology, Rheumazentrum Ruhrgebiet, Herne, Germany; Ruhr University Bochum, Bochum, Germany; Section of Rheumatology, Department of Medicine, Yale University School of Medicine, New Haven, CT, USA; Association of Women in Rheumatology, New York, NY, USA; Grace C. Wright MD PC, New York, NY, USA; Department of Medicine, Division of Rheumatology, New York University Grossman School of Medicine and Psoriatic Arthritis Center, NYU Langone Health, New York, NY, USA; AbbVie Inc, North Chicago, IL, USA; AbbVie Inc, North Chicago, IL, USA; AbbVie Inc, North Chicago, IL, USA; AbbVie Inc, North Chicago, IL, USA; Case Western Reserve University, University Hospitals Cleveland Medical Center, Cleveland, OH, USA

**Keywords:** axial spondyloarthritis, burden of disease, psychosocial outcomes

## Abstract

**Objective:**

To assess the psychosocial impact of axial spondyloarthritis (axSpA).

**Methods:**

A literature search was conducted in two stages: stage 1 included all patients with axSpA and stage 2 focused on patients with inadequate response to prior TNF inhibitor treatment. Selection criteria included population (adults with axSpA), outcomes of interest (psychosocial factors potentially impacted by axSpA, e.g. quality of life, mental health and work productivity) and context [disease-related (disease activity, pain) and -unrelated (gender, race, ethnicity, behaviour) factors potentially affecting psychosocial outcomes). Search results were categorized based on the core domains of disease activity, pain, morning stiffness, fatigue, physical function and overall functioning and health in patients with axSpA.

**Results:**

A total of 197 articles were included in this review, most of which were observational, with only one randomized controlled trial (RCT). The evidence suggests an association between greater disease burden and poorer psychosocial outcomes as well as a bidirectional relationship between disease components and psychosocial outcomes, both contributing to the overall disease burden. However, while many studies reported on psychosocial outcomes, potential relationships with disease domains or activity were not evaluated. Furthermore, there were inconsistencies across studies in how these outcomes were measured, such as the use of different tools and/or scales.

**Conclusion:**

Given the paucity of RCTs examining psychosocial outcomes in axSpA, future research should focus on standardizing assessment of psychosocial impairments experienced by patients and establishing appropriate interventions and management strategies to ensure the holistic treatment of patients with axSpA and to optimize treatment response and outcomes.

Key messages

- The psychosocial burden of axial spondyloarthritis (axSpA) includes functional, mental health, work and social impairments.
- Managing psychosocial impairments should form part of the holistic approach to axSpA treatment.
- Future axSpA research should aim to standardize psychosocial impairment assessment tools to optimize treatment response.

## Introduction

The physical symptoms of axial SpA (axSpA) and high disease activity negatively affect patients’ mental health, causing psychological distress due to chronic pain, physical disability, fatigue and overall reduced health-related quality of life (HRQoL) [1, 2]. Psychological symptoms may include fatigue, sleep disturbance, anxiety/depression and sexual dysfunction. The burden of these psychosocial factors can profoundly impact HRQoL, compromise work productivity, reduce participation in daily activities and increase demand on healthcare support [3–5].

Tumour necrosis factor inhibitors (TNFis) have demonstrated efficacy in reducing disease activity and risk of progression in patients with radiographic axSpA [r-axSpA; also known as ankylosing spondylitis (AS)] and non-radiographic axSpA (nr-axSpA). Although other biologic DMARDs (bDMARDs) and targeted synthetic DMARDs [IL-17 and Janus kinase (JAK) inhibitors, respectively] also have proven efficacy in axSpA, more data are available for TNFis. Despite the anti-inflammatory activity of these drugs, patients may experience inadequate response (IR) due to primary failure or loss of response or they may discontinue treatment due to safety concerns or intolerance [5]. The IR patient population is more likely to experience higher disease burden, diagnostic delays, progressively worse/debilitating symptoms, structural damage and compromised HRQoL [6–10]. Difficult-to-manage (D2M) axSpA is an emerging concept [8] being addressed within the Assessment of SpondyloArthritis International Society (ASAS) D2M SpA initiative. Patients with D2M axSpA may be perceived as a heterogeneous group with persistent inflammatory activity or persistent symptoms (e.g. pain) despite a reduction in inflammation. Non-response to DMARDs may be due to multiple mechanisms, including non-inflammatory and/or non-nociceptive (nociplastic) stimuli [11], as well as factors beyond those directly attributed to the disease, such as psychosocial factors, including anxiety and depression [9, 12].

The ASAS–OMERACT core outcomes set was recently updated to enable standardized evaluation across clinical studies [13]; however, the relative contribution of each domain (disease activity, pain, morning stiffness, fatigue, physical function and overall functioning and health) to psychosocial outcomes remains poorly defined.

The ASAS–EULAR recommend tailoring treatment to each individual, taking into consideration factors other than disease signs and symptoms, including comorbidities and psychosocial burden [9]. High disease activity and certain sociodemographic factors may increase the risk for mental health problems in patients with axSpA [14]. Disease activity and functionality measures (e.g. Bath indices) have been correlated to the burden of anxiety/depression in patients with axial disease [15, 16], and addressing mental comorbidities and sleep disorders is a crucial, although sometimes overlooked, aspect of comprehensive rheumatologic care.

Given the need to individualize treatment based on patients’ psychosocial impairments and comorbidities, and the limited understanding of the possible mechanisms underlying therapeutic non-response, this systematic literature review sought to elucidate the psychosocial impact of axSpA, including in patients with prior TNFi-IR. The primary objective was to investigate psychosocial outcomes affected by axSpA and other factors (disease-related and unrelated) contributing to overall disease burden in this patient population.

## Methods

### Search strategy

This systematic literature review was conducted in accordance with relevant Preferred Reporting Items for Systematic Reviews and Meta-Analyses Protocols (PRISMA) guidelines [17, 18]. PubMed was searched from 1 January 2007 through 1 May 2023 (15 years). The search scope was formally expanded to include ≈160 rheumatology journals not indexed on PubMed for psychosocial issues in axSpA; these were searched from 2018 through 2023 (5 years). Proceedings from annual meetings of key organizations (ACR, Asia-Pacific League of Associations for Rheumatology, EULAR, Japan College of Rheumatology, Pan-American League of Associations for Rheumatology) were searched from 2018 through 2023 (5 years). Older proceedings, proceedings from other conferences and other literature (e.g. reports, working papers, government documents, white papers, evaluations) were excluded. Data from non-PubMed databases (e.g. Scopus, Embase, Cochrane Library) were also excluded, as a preliminary search identified substantial overlap.

### Search stages

A systematic literature search was performed in two stages. Stage 1 included all patients with axSpA, while stage 2 focused on patients with TNFi-IR axSpA. We focused on TNFis since this drug class is considered as the first-line axSpA treatment, whereas IL-17 and JAK inhibitors are often applied as second-line treatment [5] and may have resulted in bias if included in the search.

### Study selection criteria

The protocol was developed according to the PRISMA-P guidelines [17] and the search was conducted according to PRISMA guidelines [18]. Studies were selected as per PICO criteria [19]: population (adults with axSpA), outcomes of interest (psychosocial factors affected by axSpA, such as HRQoL, mental health and work productivity) and context [disease-related (e.g. disease activity, pain) and -unrelated (e.g. gender, race, ethnicity, behaviour) factors potentially affecting the outcomes of interest]. Detailed PubMed search terms for stages 1 and 2 are provided in Supplementary Tables S1 and S2, respectively, available at *Rheumatology Advances in Practice* online. Briefly, the population included patients with axSpA in stage 1 and patients with bDMARD-IR/TNFi-IR axSpA in stage 2. Studies evaluating measures related to psychosocial outcomes listed in the search terms were included. Patients with enteropathic arthritis were included. Patients with other forms of SpA, including reactive and psoriatic arthritis, were excluded. Patients with treatment-naïve or treatment-sensitive axSpA (stage 2 only) were excluded.

During phase 1, the title of each article in the search results was evaluated; abstracts were read if inclusion could not be determined from the title alone. During phase 2, the full article of each selected study was reviewed. There were no sample size restrictions for study selection.

### Screening

#### Phase 1

Titles and abstracts retrieved during stages 1 and 2 were initially reviewed by one reviewer (M.R.) based on predetermined inclusion/exclusion criteria for the systematic literature review. Fifty percent of titles/abstracts were reviewed by a second reviewer (P.M.), with any discrepancies resolved through discussion and consensus.

#### Phase 2

Full-text articles corresponding to abstracts selected during phase 1 were reviewed by a primary reviewer (M.R.) using predefined selection criteria to ensure relevance to the systematic literature review. Articles not meeting the criteria were excluded. Fifty percent of full-text articles were reviewed by a second reviewer (P.M.), with any discrepancies resolved through discussion and consensus.

### Study assessment

The quality assessment tool used for full-text articles selected during phase 2 screening depended on the study type; the Strengthening the Reporting of Observational Studies in Epidemiology checklist [20] was used for observational studies (cohort, case–control, cross-sectional), the Cochrane risk-of-bias tool [21] for randomized control trials (RCTs) and the Joanna Briggs Institute appraisal checklist [22] for systematic reviews.

### Outcomes of interest and data extraction

Outcomes of interest evaluated in the identified studies included psychosocial outcomes affected by axSpA (e.g. anxiety/depression, functional ability, social interaction, HRQoL, fatigue, work productivity), including patient-reported outcomes (PROs); disease components (e.g. disease activity, pain); behavioural and environmental factors (e.g. BMI, smoking); and factors possibly related to healthcare inequality (e.g. gender, race, ethnicity) that may contribute to overall disease burden. These outcomes were then considered in the context of multiple ASAS-OMERACT core outcomes, which include the minimum mandatory set of domains to be assessed in all clinical trials and longitudinal observational studies evaluating a therapy in patients with axSpA [13]. The results of this literature search were categorized based on the core domains of disease activity, pain, morning stiffness, fatigue, physical function and overall functioning and health in patients with AS or nr-axSpA.

## Results

**Figure 1.**
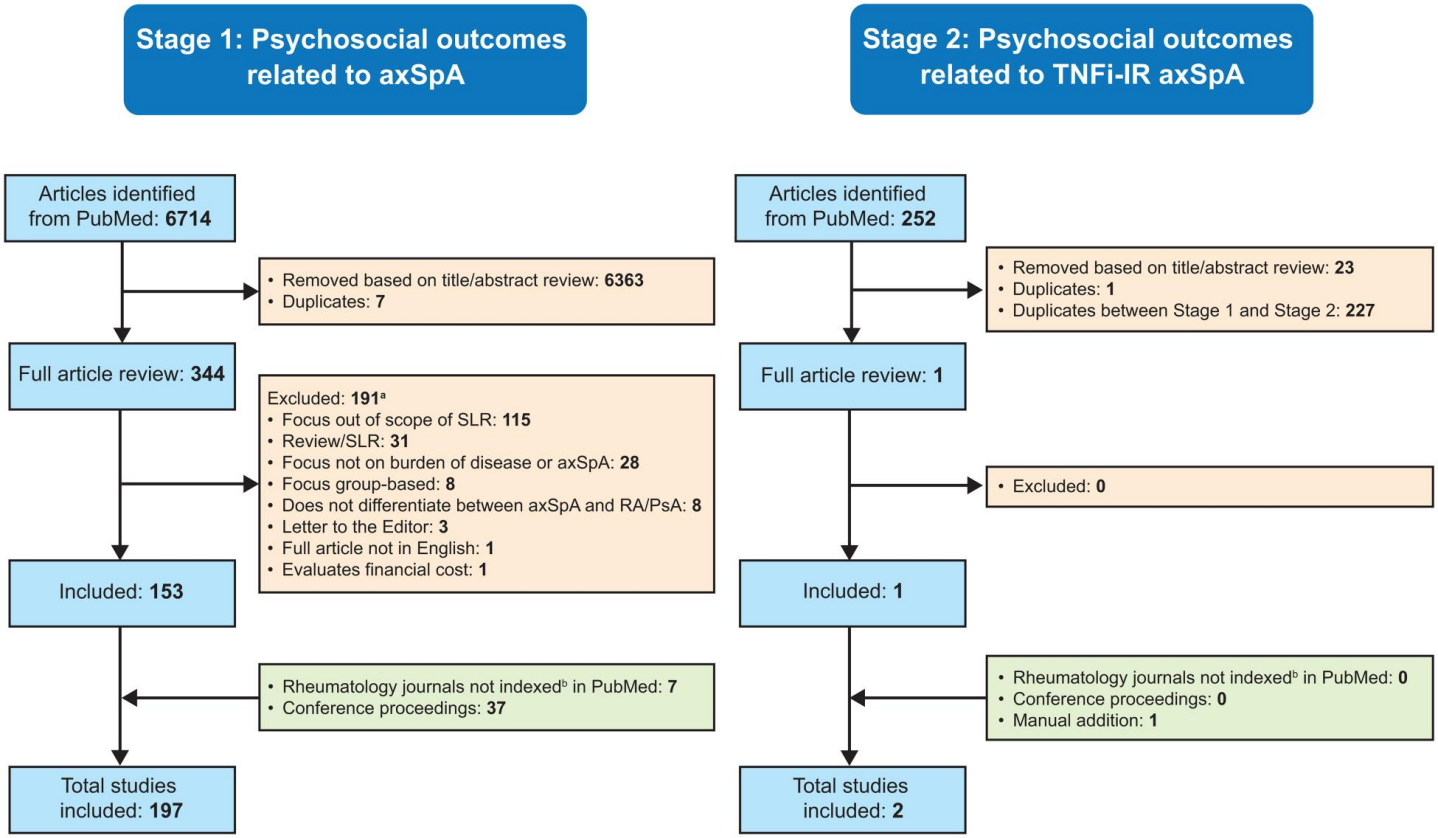
Search results. ^a^A single study may fulfil more than one criterion for exclusion. ^b^At the time of the search. SLR: systematic literature review

**Figure 2.**
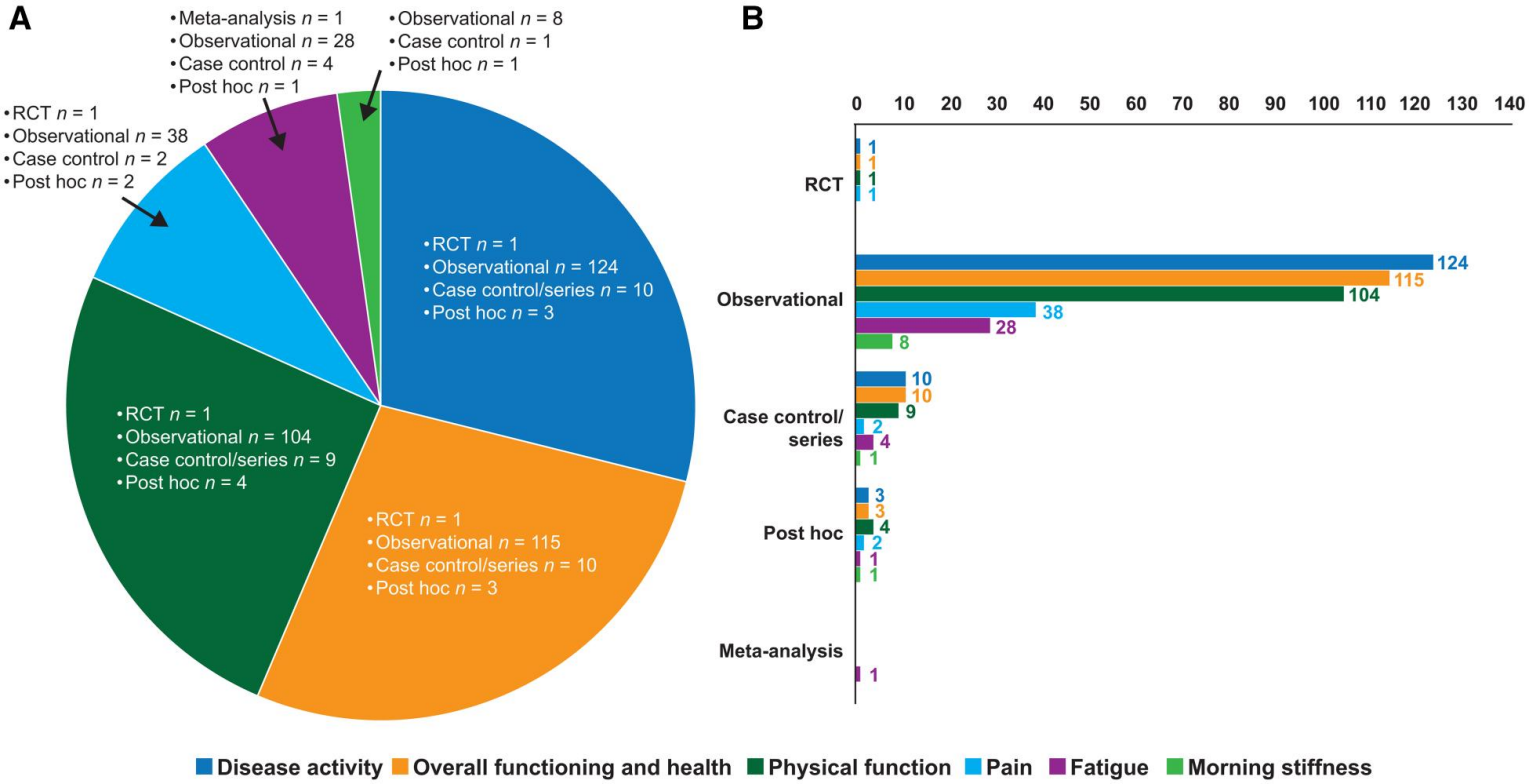
(A) Number of articles identified in the literature across six ASAS core domains and (B) types and number of studies investigating each domain

The initial search identified 6714 potential studies for review. The study selection process is summarized in Fig. 1. In total, 197 studies (Supplementary Tables S3 and S4, available at *Rheumatology Advances in Practice* online) were included in the analysis. Only one multicentre, phase 3, double-blind, placebo-controlled RCT was identified [23], which investigated improvement in PROs with adalimumab in patients with nr-axSpA and the relationship of PROs with work productivity. The remaining studies included 143 observational, 9 case–control, 4 post hoc exploratory, 3 systematic reviews/meta-analyses and 37 conference proceedings (Fig. 2). When restricting the search to patients with prior TNFi-IR, a single report of a post hoc analysis of a phase 3 trial was identified [24]; another study on the International Map of Axial Spondyloarthritis (IMAS) initiative in a large cohort of US patients with axSpA, which was not initially detected during the PubMed search, was added manually [25].

### Disease activity

In total, 138 studies (1 RCT, 124 observational, 9 case–control, 1 case series and 3 post hoc exploratory) investigated the impact of disease activity on psychosocial outcomes, including work productivity, HRQoL, overall functioning and physical and mental health (Supplementary Table S5, available at *Rheumatology Advances in Practice* online).

The RCT by Maksymowych *et al.* [23] comprised a 24-week, double-blind, placebo-controlled phase followed by an open-label extension and evaluated the impact of age, sex, physical function and HRQoL on work status and productivity. The study included 315 patients with AS receiving adalimumab for up to 3 years. Disease activity (per the BASDAI) was significantly associated with work presenteeism; patients’ global assessment of disease activity [visual analogue scale (VAS)] was also independently associated with working status (*P* < 0.001). Findings from larger observational studies (>500 patients) support an inverse association between disease activity and work productivity. A cross-sectional, UK-based study [26] including 612 patients of working age, showed that worse disease activity was associated with worse work-related outcomes, particularly absenteeism and reduced productivity. An observational study in Germany, including 770 patients with axSpA, also reported that higher disease activity was associated with impaired work productivity [27]. Another observational study in Spain, including 653 patients with axSpA, reported that patients who were of working age but unemployed partially attributed their employment status to their disease activity [28]. A cross-sectional study across 13 European countries, including 2695 patients with axSpA, found that higher disease activity (per the BASDAI) was an independent determinant of work-related problems [4]. However, these findings were not universal; e.g. a cross-sectional study in Spain, including 699 patients with AS, found no association between BASDAI scores and work disability [29].

An observational study [30] including 962 patients with AS found a strong correlation between disease activity and AS-related QoL (ASQoL), with a BASDAI score ≥4 being significantly related to poor QoL outcomes (*P* < 0.001). Furthermore, while both the Axial Spondyloarthritis Disease Activity Score with CRP (ASDAS-CRP) and BASDAI were significantly associated with disability (per the HAQ for AS) in a study of early axSpA, the ASDAS-CRP was the greatest predictor of disability outcomes [31]. In a post hoc, cross-sectional analysis of an RCT evaluating recombinant infliximab in AS, disease activity was independently associated with the 36-item Short Form (SF-36) physical component score (PCS) and mental component score (MCS). Additionally, disease activity was one of two determinants of HRQoL [32].

Greater disease activity was also independently associated with poor sexual health in AS (*P* ≤ 0.05) [33], poor sleep in axSpA (*P* < 0.00001) [34] and increased likelihood of driving difficulties in AS across three domains: dynamic scenarios, crossing traffic and the physical act of driving [35]. Moreover, the BASDAI was significantly worse in women *vs* men in a study assessing sex differences in axSpA [36] and was separately associated with anxiety and depression [37].

Of the two studies that included patients with TNFi-IR, the post hoc analysis of the ABILITY-1 trial (NCT00939003) compared outcomes for adalimumab responders and non-responders among patients with nr-axSpA [24]. The analysis found that improvements in disease activity with adalimumab [ASDAS inactive disease (<1.3), clinically meaningful improvement (≥1.1-point decrease) and major improvement (≥2-point decrease)] were associated with significantly greater improvements across most PROs, including the HAQ for Spondyloarthropathies (HAQ-S), SF-36 PCS, presenteeism, overall work impairment and activity impairment [24]; non-responders to treatment had significantly fewer improvements *vs* responders in the HAQ-S, SF-36 PCS and social participation (*P* < 0.0001) [24]. In the IMAS self-reported survey study [25], moderate to high disease activity per the BASDAI was associated with increased psychological distress and work impairment *vs* low disease activity.

### Pain

In total, 43 studies investigated pain in axSpA and its impact on psychosocial outcomes, including HRQoL and work productivity. These comprised 1 RCT [23], 1 post hoc exploratory analysis of pooled data from four published RCTs [38], 38 observational studies [26, 30, 33, 35, 39–72], 1 post hoc analysis of registry data [73] and 2 case–control studies [74, 75] (Supplementary Table S6, available at *Rheumatology Advances in Practice* online).

The RCT by Maksymowych *et al.* [23] evaluated total back pain (VAS), nocturnal pain (VAS) and the SF-36 bodily pain domain. Nocturnal pain was significantly and independently associated with working status (*P* < 0.001). In addition, all assessed PRO measures of pain were weakly correlated with work absenteeism, while work presenteeism was moderately correlated with the SF-36 bodily pain domain.

A post hoc exploratory analysis [38] presented data from four RCTs including 1283 patients with AS treated with either etanercept, sulfasalazine or placebo and evaluated the impact of biologic sex on disease burden. Total and nocturnal back pain (VAS) were evaluated in these studies, with women demonstrating a higher burden of pain and less improvement with treatment than men. Across observational studies with cohorts of ≥500, higher levels of total and nocturnal back pain were associated with higher levels of inflammatory markers and disease activity [59], work absenteeism and reduced work productivity [55, 68]. Women and older patients appeared to experience worse pain and HRQoL compared with men [61, 68].

An observational study reported a significant correlation (*P* < 0.001) between pain and ASQoL [30], while another reported an association between pain and driving difficulties [35]. Higher pain levels were associated with reduced happiness (SF-36); problems with mobility, self-care and usual activities; and anxiety/depression [EuroQoL 3-level 5-domain questionnaire (EQ-5D-3L)] [68]. Smaller observational studies supported these findings and also reported associations between pain and sexual dysfunction [51], sleep disturbance [74] and neuropsychological phenomena such as alexithymia [48].

### Morning stiffness

In total, 10 studies investigated the impact of morning stiffness on psychosocial outcomes in patients with axSpA. These included eight observational studies (most of which were cross-sectional) [40, 43, 46, 58, 70, 71, 76, 77], one post hoc analysis of registry data [73] and one case–control study [74] (Supplementary Table S7, available at *Rheumatology Advances in Practice* online).

A cross-sectional study reported that sexual dysfunction was associated with depression and limited joint mobility (BASMI) in men with AS but was not significantly associated with other disease parameters, including morning stiffness [76]. However, in a separate study of women, the duration of morning stiffness was negatively correlated with sexual desire (*P* < 0.05) compared with healthy controls [77], suggesting a complicated and multifactorial causation of sexual dysfunction in AS. A multicentre, cross-sectional study [46] found that morning stiffness duration was significantly associated with increased Hospital Anxiety and Depression Scale (HADS) scores. Morning stiffness, also a significant factor underpinning poor Multidimensional Assessment of Fatigue scores [40], was strongly correlated with poor ASQoL outcomes in patients with peripheral involvement (*P* < 0.05) [71] and, when prolonged, was moderately correlated with poorer HRQoL as measured by the SF-36 (*P* < 0.001) [58]. Morning stiffness was not significantly related to BASMI in a small study examining the relationship between spinal mobility and QoL in AS [70]. A post hoc analysis of registry data investigating patients’ priorities in dimensions of health across inflammatory rheumatic diseases found morning stiffness among the top three priority health issues for improvement in 44.3% of patients [73].

### Fatigue

In total, 34 studies investigated fatigue in axSpA and its relationship with other disease-related factors, including disease activity, and psychosocial outcomes such as HRQoL, mental health and work productivity. The studies included 1 systematic review and meta-analysis [78], 1 post hoc analysis of registry data [73], 28 observational [30, 34, 35, 39, 40, 42, 43, 47, 49, 53, 55, 57, 62, 68, 71, 79–91] and 4 case–control studies [92–95] (Supplementary Table S8, available at *Rheumatology Advances in Practice* online).

A key objective of the systematic review and meta-analysis, which included 30 studies (7893 patients in total), was to identify factors associated with fatigue in patients with axSpA. A significant association (*P* < 0.05) between increased fatigue and poorer ASQoL was identified [78]. A relationship between fatigue and QoL outcomes was also seen among studies of different sizes, including large observational studies (>500 patients) [30, 79]. Bedaiwi *et al.* [79] reported that patients with axSpA and severe fatigue had significantly impaired QoL across numerous assessments (SF-36 MCS, SF-36 PCS, ASQoL, EQ-5D and HAQ; all *P* < 0.0001 *vs* low fatigue). Assessing fatigue frequency and severity is crucial for understanding its impact, as 79% of patients with AS with both frequent and severe fatigue at baseline reported no change in their fatigue level at 6 months [82]. Patients with severe and frequent or severe but not frequent fatigue also had worse associated psychosocial outcomes and were more likely to use TNFi *vs* those with frequent fatigue only; however, the clinical relevance of this observation was reduced due to the limited proportion of patients receiving TNFi [82].

One large observational study reported that high fatigue levels were associated with poor sleep among patients with axSpA [34]. Additionally, patients with AS receiving TNFi or anti-IL-17A therapy in a smaller multicentre study demonstrated significant associations between Functional Assessment of Chronic Illness Therapy – Fatigue and Insomnia Severity Index scores (*P* < 0.0001 and *P* = 0.0001, respectively) [89]. Small studies also found significant associations between fatigue and sleep disturbance in AS, as well as depression, anxiety, worse physical and mental QoL, worse disease-related outcomes and reduced functional capacity [39, 53, 91, 94]. From a patient perspective, the factor most strongly associated with fatigue was pain; there was no strong association of fatigue with anxiety, motivation or depression [47].

Increased fatigue severity in AS was associated with greater functional disability and reduced general well-being, higher disease activity and increased enthesitis severity [83]. Connolly *et al.* [49] found that severe fatigue in AS was associated with reduced occupational participation in productivity and leisure, reduced QoL, more active disease, higher pain and reduced physical capacity, with significant differences in outcomes *vs* low fatigue. High fatigue levels in axSpA were also associated with negative work-related outcomes, including increased absenteeism, presenteeism, productivity loss and daily activity impairment [55]. Gossec *et al.* [80] suggested that fatigue in early axSpA may be a fundamental component of the underlying disease process. Fatigue was significantly correlated with BASFI scores in AS [62] and increased neuropathic pain [57] and inflammatory bowel disease [87] in axSpA. Fatigue was also shown to be a significant factor negatively impacting sexual function in men with AS [86].

### Physical function

In total, 118 studies [1 RCT, 104 observational studies, 4 post hoc exploratory analyses (of which 3 were of RCTs), 8 case–control studies and 1 retrospective case series] investigated the relationship between physical function and psychosocial outcomes in axSpA (Supplementary Table S9, available at *Rheumatology Advances in Practice* online).

In the Maksymowych *et al.* RCT [23], measures of poor physical function in AS were significantly correlated with working status and presenteeism, suggesting a causal effect of compromised physical function on patients’ reduced ability to work.

Across 20 large observational studies (>500 patients), greater impairment of physical function (commonly measured using the BASFI) was associated with female sex [96], reduced HRQoL [30], unemployment [26, 28, 97], work disability [29], impaired work productivity [27], increased healthcare costs and loss of work productivity relating to AS [98], increased risk for mental health disorders, poorer mental health outcomes [2, 37], depression (patient- and claims-reported data) [99], depressive symptoms [100], higher frequency and severity of fatigue [82], negative impact on sexual relationships [33] and poor sleep [34]. While no differences in physical function (per the BASFI) were observed between sexes in a large study [36], a smaller study [61] in 498 patients found that women experienced a greater disease burden, including impaired physical function (*P* < 0.01).

In a large post hoc analysis of four RCTs, in which patients with AS were treated using either etanercept, sulfasalazine or placebo, women experienced significantly smaller improvements in physical functioning *vs* men at 12-week efficacy assessments (*P* < 0.05) [38]. In a smaller, post hoc cross-sectional analysis of an RCT evaluating recombinant infliximab in AS, physical function was independently associated with SF-36 PCS and MCS. Additionally, in a proposed stratified model, physical function was one of two determinants (disease activity was the other) of HRQoL outcomes [32], suggesting that both physical function improvement and disease activity control are needed to optimize HRQoL in patients with AS. However, as the patient cohort was from an RCT, these findings may be less generalizable owing to more severe disease in this population [32]. Lastly, the post hoc analysis of the ABILITY-1 trial demonstrated that a ≥40% improvement and 20 U absolute improvement from baseline and ASDAS responses in patients with nr-axSpA receiving bDMARDs were associated with clinically meaningful improvements in physical function (BASFI, SF-36 PCS) [24]. The IMAS study also demonstrated the substantial impact of the disease on daily function, including both physical and psychological burden [25].

Several outcomes seen in large studies were also observed in smaller studies [49, 58, 64, 95, 101–107]. The BASFI was a significant and robust predictor of EQ-5D outcomes [104] and was significantly related to all four physical domains and two mental health domains (vitality and mental health) of the SF-36 [62]. Poor functional ability was associated with sexual impairment in AS, especially in women [77], and physical function was impaired in early-stage axSpA in a small patient cohort [108]. Patients with AS were also shown to have worse overall HRQoL, which may be attributed to the psychosocial impact of high-burden disease [109].

### Overall functioning and health

In total, 129 studies investigated disease impact on overall HRQoL, functioning and health, including mental health. The most commonly used tools included the ASQoL, HADS, HAQ Disability Index and SF-36. The identified studies comprised 1 RCT [23], 115 observational studies [2, 4, 14, 23, 25–31, 33–35, 37, 39–44, 46–55, 57–66, 68–71, 76, 77, 79–88, 90, 91, 97, 99, 100, 102–105, 107–155], 3 post hoc analyses [24, 32, 38, 73], 9 case–control studies [74, 75, 92–95, 156–158] and 1 case series [159] (Supplementary Table S10, available at *Rheumatology Advances in Practice* online).

In the Maksymowych *et al.* RCT [23], work absenteeism was weakly correlated with all PRO scores; presenteeism was moderately correlated with ASQoL, SF-36 bodily pain domain, BASFI, patient’s global assessment of disease activity (all *P* < 0.001), SF-36 PCS and nocturnal pain (both *P* < 0.005). One post hoc analysis of registry data [73] reported that pain (57%), physical function (19.4%), fatigue (11.8%), morning stiffness (8.9%), sleep (8%) and emotional well-being (2.1%) were prioritized by patients as health issues for improvement; sleep problems were of higher priority for improvement for patients with AS compared with other inflammatory arthropathies. One post hoc analysis of a double-blind, placebo-controlled clinical trial, proposing a stratified model for health outcomes in AS demonstrated that HRQoL is determined by physical function and disease activity, physical function is determined by spinal mobility and disease activity and spinal mobility is determined by structural damage and spinal inflammation [32].

## Discussion

This systematic literature review identified the impact of multiple ASAS-OMERACT core domains [13] on several psychosocial outcomes, including work performance, HRQoL, sleep quality, sexual functioning and mental health. A large number of studies were identified and 197 were included in the review. The majority were observational and many, while reporting on psychosocial outcomes, did not explore potential relationships with disease domains or activity. Consequently, while the current evidence base suggests an association between greater disease burden and poorer psychosocial outcomes, the direction and magnitude of this influence may vary. In addition, although the outcomes of interest in this systematic literature review were the psychosocial factors affected by axSpA, it may not always be possible to clearly delineate factors and outcomes, as there is a bidirectional relationship between disease components and psychosocial outcomes, as well as between different psychosocial outcomes and PROs, each contributing to overall disease burden by forming parts of a cycle. Therefore, the negative impact of psychosocial factors should also be taken into account when treating patients with axSpA, as the findings suggest that managing these factors results in improved outcomes and should be part of the holistic approach to the treatment of axSpA.

The lack of consistency across studies in how these outcomes were measured and the assessment of their impact on axSpA-specific disease domains represents an important knowledge gap, particularly for patients with prior TNFi-IR, for whom similar data are lacking. These patients are more difficult to treat, highlighting the importance of evaluating and addressing psychosocial disease aspects, as these may negatively impact treatment response. Numerous factors likely contribute to an apparent non-response to therapy, including those related to the disease itself, and external factors influencing self-perceptions of symptomatology and treatment-related outcomes. Indeed, current disease activity scores used in axSpA, such as the BASDAI and BASFI, are subjective, and so are likely influenced by the broader perception of health and well-being. For example, pain catastrophizing (PC; the tendency to describe the severity and impact of pain in more exaggerated terms) is best explained by biologic subjective measures, and PC scores ≥4 are associated with inferior HRQoL in inflammatory arthritides, including axSpA [160]. When patients present with non-response to biologic therapy, they are often cycled to alternate therapy options, based on a treat-to-target strategy [10, 161]. Alternatively, partial responders with residual signs and symptoms may remain on suboptimal treatment for different reasons, including fear of losing what gains they have made in terms of perceived disease control, or other unaddressed care-related concerns. Without insights into the potential contribution of psychosocial factors that may be partially impeding response, and evaluation of appropriate interventions to manage such factors, cycling to alternative therapies may not be the optimal approach for some patients.

Although certain psychosocial outcomes (e.g. work productivity, HRQoL, mental health) appeared to be more frequently evaluated, others (e.g. income, education level, marital status, caregiver burden, etc) were infrequently or not evaluated, further underlining the current knowledge gaps. In addition, despite increasing interest in this topic in recent years, most studies are observational. Furthermore, although psychosocial outcomes may be reported, these are usually not the main study focus. There is also a relative lack of published data on the effect of different treatments and comorbidities on psychosocial burden in patients with axSpA, which are areas that require additional research.

The insights provided by the present systematic literature review are particularly relevant for psychosocial impairments for which effective interventions are available, such as anxiety/depression and poor sleep quality. Patients with AS experience significantly higher rates of anxiety and depression than the general population [2, 16], and patients with axSpA frequently experience sexual problems that may be further associated with poor mental health [33, 156]. Identification, assessment and appropriate management of these issues might, in addition to improving overall health, positively impact patients’ subjective perception of their axSpA-related burden, allowing them to continue treatment that effectively addresses their underlying pathology.

## Conclusion

The paucity of RCTs examining psychosocial outcomes indicates that these are not routinely evaluated in clinical trials. There is also a lack of consistency across observational studies in how these outcomes are measured. Future research should focus on standardizing the assessment of psychosocial impairments experienced by patients with axSpA, as well as the optimal timing (e.g. routine evaluation at treatment initiation or at the time of non-response).

More work is needed to better understand the journey of patients with advanced axSpA who have not responded to prior treatment with regard to the psychosocial burden they experience. This burden should be addressed with appropriate interventions to ameliorate perceived IR to treatment in this patient population.

## Supplementary Material

rkaf063_Supplementary_Data

## Data Availability

AbbVie is committed to responsible data sharing regarding the clinical trials we sponsor. This includes access to anonymized, individual and trial-level data (analysis datasets), as well as other information (e.g. protocols, clinical study reports or analysis plans), as long as the trials are not part of an ongoing or planned regulatory submission. This includes requests for clinical trial data for unlicensed products and indications. These clinical trial data can be requested by any qualified researchers who engage in rigorous, independent scientific research and will be provided following review and approval of a research proposal and statistical analysis plan and execution of a data sharing agreement. Data requests can be submitted at any time after approval in the USA and Europe and after acceptance of this manuscript for publication. The data will be accessible for 12 months, with possible extensions considered. For more information on the process or to submit a request, please visit the following link: https://www.abbvieclinicaltrials.com/hcp/data-sharing.
