# Supplementary material for: Psychosocial burden of axial spondyloarthritis and impact of different disease domains: a systematic literature review
Source: Rheumatol Adv Pract. 2025 May 26;9(3):rkaf063. doi: 10.1093/rap/rkaf063 (PMC12202762; doi:10.1093/rap/rkaf063)
Supplement: rkaf063_Supplementary_Data [file rkaf063_supplementary_data.zip › rkaf063_supplementary_data.docx]

**Psychosocial burden of axial spondyloarthritis and impact of different disease domains: a systematic literature review**

**Denis Poddubnyy^1,2,^*, Uta Kiltz^3,4^, Abhijeet Danve^5^, Grace Wright^6,7^, Rebecca Haberman^8^, Ana Biljan^9^, Jerry Clewell^9^, Jamie Urbanik^9^, Heather Jones^9^, Marina Magrey^10^**

^1^Division of Rheumatology, Department of Medicine, University of Toronto, Toronto, Canada

^2^Department of Gastroenterology, Infectious Diseases and Rheumatology, Charité-Universitätsmedizin Berlin, Berlin, Germany
^3^Rheumazentrum Ruhrgebiet, Rheumatology, Herne, Germany
^4^Ruhr University Bochum, Bochum, Germany
^5^Section of Rheumatology, Department of Medicine, Yale University School of Medicine, New Haven, CT, USA
^6^Association of Women in Rheumatology, New York, NY, USA
^7^Grace C Wright MD PC, New York, NY, USA
^8^Department of Medicine, Division of Rheumatology, New York University Grossman School of Medicine and Psoriatic Arthritis Center, NYU Langone Health, New York, NY, USA
^9^AbbVie Inc., North Chicago, IL, USA
^10^Case Western Reserve University, MetroHealth Medical Center, Cleveland, OH, USA

*Correspondence to: Denis Poddubnyy, Division of Rheumatology, Department of Medicine, University Health Network and University of Toronto, 399 Bathurst St., Toronto, ON M5T 2S6, Canada.

E-mail: [denis.poddubnyy@uhn.ca](mailto:denis.poddubnyy@uhn.ca)

ORCID iD: 0000-0002-4537-6015

**Supplementary materials**

| Table S1. Stage 1 PubMed search terms | |
| --- | --- |
| Population | “non-radiographic axial spondyloarthritis” [MeSH] **OR** “nr-axial spondyloarthritis” **OR** “non-radiographic axSpA” **OR** “nrAxSpA” **OR** “radiographic axial spondyloarthritis” **OR** “radiographic axSpA” **OR** “r-axSpA” **OR** “raxSpA” **OR** “spondylitis, ankylosing” [MeSH] **OR** “AS” **OR** “enteropathic arthritis” **OR** “enteroarthritis” **OR** “inflammatory bowel disease associated arthritis” |
| Psychosocial factors  (outcomes of interest) | “psychology” [MeSH] **OR** “cognitive” **OR** “social support” [MeSH] **OR** “burden” **OR** “caregiver burden” [MeSH] **OR** “fear” [MeSH] **OR** “worry” **OR** “mental health” [MeSH] **OR** “impact” **OR** “loneliness” [MeSH] **OR** “quality of life” [MeSH] **OR** “QoL” **OR** “depression” [MeSH] **OR** “dysthymic disorder” [MeSH] **OR** “MDD” **OR** “bipolar disorder” [MeSH] **OR** “anxiety” [MeSH] **OR** “GAD” **OR** “anti-depressant” **OR** “psychiatric” **OR** “stress” **OR** “pain” [MeSH] **OR** “psychological distress” [MeSH] **OR** “nocturnal” **OR** “sleep initiation and maintenance disorders” [MeSH] **OR** “affect” [MeSH] **OR** “social stigma” [MeSH] **OR** “stigmatization” **OR** “impairment” **OR** “suicidal ideation” [MeSH] **OR** “affective symptoms” [MeSH] **OR** “anger” [MeSH] **OR** “sexual dysfunction” **OR** “self-consciousness” **OR** “disorder” **OR** “somatoform disorder” [MeSH] **OR** “adaptation, psychological” [MeSH] **OR** “sexual dysfunction” **OR** “relationship difficulties” **OR** “behavioural” **OR** “panic” [MeSH] **OR** “feeding and eating disorders” [MeSH] **OR** “obsessive behavior” [MeSH] **OR** “agoraphobia” [MeSH] **OR** “phobia, social” [MeSH] **OR** “helplessness” **OR** “hopelessness” **OR** “sadness” [MeSH] **OR** “attention deficit disorder with hyperactivity” [MeSH] **OR** “avoidance learning” [MeSH] **OR** “embarrassment” **OR** “schizophrenia” [MeSH] **OR** “substance-related disorders” **OR** “smoking” **OR** “body mass index” [MeSH] |
| Context (healthcare disparity factors) | “healthcare disparities” [MeSH] **OR** “disparities” **OR** “disparity” **OR** “health equity” [MeSH] **OR** “racial” **OR** “racism” [MeSH] **OR** “race factors” [MeSH] **OR** “ethnic and racial minorities” **OR** “Black people” [MeSH] **OR** “Black or African American” [MeSH] **OR** “Hispanic” [MeSH] **OR** “Latino” [MeSH] **OR** “Asian people” [MeSH] **OR** “Asian” [MeSH] **OR** “Pacific island people” [MeSH] **OR** “Indigenous Peoples” [MeSH] **OR** “American Indian or Alaska Native” [MeSH] **OR** “First Nations” [MeSH] **OR** “minority groups” [MeSH] **OR** “sexism” [MeSH] **OR** “socioeconomic factors” [MeSH] **OR** “socioeconomic disparities in health” [MeSH] |

| Table S2. Stage 2 PubMed search terms | |
| --- | --- |
| Population |  |
| Part 1 | “tumor necrosis factor inhibitors” [MeSH] |
| Part 2 | “treatment failure” [MeSH] **OR** “treatment failed” **OR** “failed” **OR** “intolerant” **OR** “inadequate response” **OR** “prior use” **OR** “previous use” **OR** “treatment experienced” **OR** “TNF-ir” **OR** “TNF-it” |
| Part 3 | “non-radiographic axial spondyloarthritis” [MeSH] **OR** “nr-axial spondyloarthritis” **OR** “non-radiographic axSpA” **OR** “nrAxSpA” **OR** “radiographic axial spondyloarthritis” **OR** “radiographic axSpA” **OR** “r-AxSpA” **OR** “rAxSpA” **OR** “spondylitis, ankylosing” [MeSH] **OR** “AS” **OR** “enteropathic arthritis” **OR** “enteroarthritis” **OR** “inflammatory bowel disease associated arthritis” |
| Psychosocial factors  (outcomes of interest) | “psychology” [MeSH] **OR** “cognitive” **OR** “social support” [MeSH] **OR** “burden” **OR** “caregiver burden” [MeSH] **OR** “fear” [MeSH] **OR** “worry” **OR** “mental health” [MeSH] **OR** “impact” **OR** “loneliness” [MeSH] **OR** “quality of life” [MeSH] **OR** “QoL” **OR** “depression” [MeSH] **OR** “dysthymic disorder” [MeSH] **OR** “MDD” **OR** “bipolar disorder” [MeSH] **OR** “anxiety” [MeSH] **OR** “GAD” **OR** “anti-depressant” **OR** “psychiatric” **OR** “stress” **OR** “pain” [MeSH] **OR** “psychological distress” [MeSH] **OR** “nocturnal” **OR** “sleep initiation and maintenance disorders” [MeSH] **OR** “affect” [MeSH] **OR** “social stigma” [MeSH] **OR** “stigmatization” **OR** “impairment” **OR** “suicidal ideation” [MeSH] **OR** “affective symptoms” [MeSH] **OR** “anger” [MeSH] **OR** “sexual dysfunction” **OR** “self-consciousness” **OR** “disorder” **OR** “somatoform disorder” [MeSH] **OR** “adaptation, psychological” [MeSH] **OR** “sexual dysfunction” **OR** “relationship difficulties” **OR** “behavioural” **OR** “panic” [MeSH] **OR** “feeding and eating disorders” [MeSH] **OR** “obsessive behavior” [MeSH] **OR** “agoraphobia” [MeSH] **OR** “phobia, social” [MeSH] **OR** “helplessness” **OR** “hopelessness” **OR** “sadness” [MeSH] **OR** “attention deficit disorder with hyperactivity” [MeSH] **OR** “avoidance learning” [MeSH] **OR** “embarrassment” **OR** “schizophrenia” [MeSH] **OR** “substance-related disorders” |
| Context (healthcare disparity factors) | “healthcare disparities” [MeSH] **OR** “disparities” **OR** “disparity” **OR** “health equity” [MeSH] **OR** “racial” **OR** “racism” [MeSH] **OR** “race factors” [MeSH] “ethnic and racial minorities” **OR** “Black people” [MeSH] **OR** “Black or African American” [MeSH] **OR** “Hispanic or Latino” [MeSH] **OR** “Asian people” [MeSH] **OR** “Asian” [MeSH] **OR** “Pacific island people” [MeSH] **OR** “Indigenous Peoples” [MeSH] **OR** “American Indian or Alaska Native” [MeSH] **OR** “First Nations” **OR** “minority groups” [MeSH] **OR** “sexism” [MeSH] **OR** “socioeconomic factors” [MeSH] **OR** “socioeconomic disparities in health” [MeSH] |

| Table S3. Articles included in the SLR sourced from PubMed and non-PubMed indexed journals (*N* = 160) | | | | | | | |
| --- | --- | --- | --- | --- | --- | --- | --- |
| Publication, year [Ref] | **Study type** | **Location** | | **Number of patients** | | **Disease** | |
| A. Studies included in PubMed (*n* = 153) | | | | | | | |
| Aggarwal R, Malaviya AN. Clin Rheumatol, 2009 [1] | Single-center, prospective cohort | | India | | 70 | | AS |
| Aissaoui N, et al. Rheumatol Int, 2012 [2] | Cross-sectional | | Morocco | | 110 | | AS |
| Alkan BM, et al. Mod Rheumatol, 2013 [3] | Cross-sectional | | Turkey | | 110 (+ 40 healthy volunteers) | | AS |
| Ariza-Ariza R, et al. J Rheumatol, 2009 [4] | Cross-sectional | | Spain | | 699 | | AS |
| Atar E, Askin A. Int J Rheum Dis, 2020 [5] | Cross-sectional | | Turkey | | 80 | | AS |
| Aykurt Karlıbel İ, et al. Aging Male, 2019 [6] | Prospective, observational cohort | | Turkey | | 67 | | AS |
| Aykurt Karlıbel I, Kasapoğlu Aksoy M. Ir J Med Sci, 2023 [7] | Cross-sectional | | Turkey | | 82 (+ 40 healthy volunteers) | | axSpA |
| Bakland G, et al. J Rheumatol, 2011 [8] | Single-center, prospective, observational cohort | | Norway | | 360 | | AS |
| Bal S, et al. Rheumatol Int, 2011 [9] | Single-center, prospective, observational | | Turkey | | 37 (+ 67 healthy controls) | | AS |
| Bandinelli F, et al. Clin Rheumatol, 2016 [10] | Single-center, retrospective cohort | | Italy | | 135 | | AS |
| Batmaz İ, et al. Rheumatol Int, 2013 [11] | Single-center,  cross-sectional | | Turkey | | 80 (+ 52 controls) | | AS |
| Baysal O, et al. Rheumatol Int, 2011 [12] | Multicenter,  cross-sectional | | Turkey | | 243 (+ 118 healthy controls) | | AS |
| Bedaiwi M, et al. J Rheumatol, 2015 [13] | Longitudinal cohort | | Canada | | 681 | | axSpA |
| Bodur H, et al. Qual Life Res, 2011 [14] | Single-center  cross-sectional | | Turkey | | 962 | | AS |
| Boonen A, et al. J Rheumatol, 2015 [15] | Prospective, multicenter,  open-label, observational cohort | | Belgium | | 80 | | AS |
| Boonen A, et al. Rheumatology (Oxford), 2018 [16] | Prospective, multicenter, open-label, observational cohort | | Belgium | | 75 | | AS |
| Brophy S, et al. Semin Arthritis Rheum, 2013 [17] | Prospective, multicenter, observational | | UK | | 385 | | AS |
| Cakar E, et al. Clin Rheumatol, 2007 [18] | Double-center,  cross-sectional | | Turkey | | 53 | | AS |
| Cakar E, et al. Clin Rheumatol, 2009 [19] | Prospective, observational | | Turkey | | 121 | | AS |
| Carvalho PD, et al. Arthritis Care Res (Hoboken), 2022 [20] | Prospective, multicenter, observational cohort | | France | | 644 | | Early axSpA |
| Cengiz G, et al. Int J Rheum Dis, 2023 [21] | Single-center,  cross-sectional | | Turkey | | 110 | | axSpA |
| Chen CH, et al. Int J Rheum Dis, 2020 [22] | Single-center,  cross-sectional | | Taiwan | | 105 | | AS |
| Chen CH, et al. Clin Rheumatol, 2013 [23] | Single-center,  cross-sectional | | Taiwan | | 75 | | AS |
| Chen HH, et al. PLoS One, 2020 [24] | Single-center,  cross-sectional | | Taiwan | | 307 | | AS |
| Chen MH, et al. Clin Rheumatol, 2018 [25] | Multicenter, retrospective | | Taiwan | | 120 | | AS |
| Chung DXY, et al. BMJ Open, 2023 [26] | Single-center,  cross-sectional | | Singapore | | 296 | | axSpA |
| Chung HY, et al. Ann Rheum Dis, 2012 [27] | Multicenter,  cross-sectional | | France | | 647 | | axSpA |
| Connolly D, et al. Occup Ther Int, 2019 [28] | Single-center,  cross-sectional | | Ireland | | 50 | | AS |
| Cunha RN, et al. ARP Rheumatol, 2022 [29] | Multicenter,  cross-sectional | | Portugal | | 1995 | | axSpA |
| D'Angelo S, et al. Clin Exp Rheumatol, 2019 [30] | Multicenter, prospective, observational | | Italy | | 512 | | axSpA |
| D'Angelo S, et al. Clin Exp Rheumatol, 2021 [31] | Multicenter, prospective, observational | | Italy | | 512 | | axSpA |
| Demir SE, et al. Rheumatol Int, 2013 [32] | Single-center,  cross-sectional | | Turkey | | 23 (+ 27 healthy controls) | | AS |
| Dhakad U, et al. Int J Rheum Dis, 2015 [33] | Single-center, prospective,  case-control study | | India | | 100 (+ 100 healthy controls) | | AS |
| Dincer U, et al. Tohoku J Exp Med, 2007 [34] | Single-center,  cross-sectional | | Turkey | | 36 (+ 34 controls) | | AS |
| Dincer U, et al. Rheumatol Int, 2007 [35] | Single-center,  cross-sectional | | Turkey | | 65 (+ 45 healthy controls) | | AS |
| Dülger S, et al. J Clin Rheumatol, 2019 [36] | Single-blind, controlled, observational | | Turkey | | 92 | | AS |
| Durcan L, et al. J Rheumatol, 2012 [37] | Single-center,  cross-sectional | | Ireland | | 46 | | AS |
| Durmus D, et al. Compr Psychiatry, 2015 [38] | Single-center,  cross-sectional | | Turkey | | 80 (+ 80 healthy controls) | | AS |
| Elolemy G, et al. Curr Rheumatol Rev, 2020 [39] | Multicenter,  cross-sectional | | Kuwait and Saudi Arabia | | 47 | | AS |
| Espahbodi S, et al. Clin Exp Rheumatol, 2017 [40] | Single-center,  cross-sectional | | UK | | 490 | | axSpA |
| Essers I, et al. Rheumatology (Oxford), 2016 [41] | Multicenter, prospective cohort | | The Netherlands | | 90 | | AS |
| Fernández-Carballido C, et al. Arthritis Care Res (Hoboken), 2020 [42] | Multicenter,  cross-sectional | | Spain | | 738  (+ 721 with PsA) | | AS |
| Fernández-Carballido C, et al; Esperanza Study Group. Arthritis Care Res (Hoboken), 2017 [43] | Multicenter,  cross-sectional | | Spain | | 259 | | Early axSpA |
| Fongen C, et al. Musculoskeletal Care, 2015 [44] | Single-center,  cross-sectional | | Norway | | 148 (+ 133 controls) | | AS |
| Forejtová S, et al. Clin Rheumatol, 2008 [45] | Single-center,  cross-sectional | | Czech Republic | | 1001 | | AS |
| Frede N, et al. RMD Open, 2023 [46] | Single-center, retrospective,  cross-sectional | | Austria | | 162  (+ 168 with PsA) | | axSpA |
| Garrido-Cumbrera M, et al. Arthritis Care Res (Hoboken), 2021 [47] | Multicenter,  cross-sectional | | 13 European countries | | 2695 | | axSpA |
| Garrido-Cumbrera M, et al.  J Rheumatol, 2022 [48] | Cross-sectional | | Spain | | 653 | | axSpA |
| Garrido-Cumbrera M.  J Rheumatol, 2019 [49] | Cross-sectional | | Spain | | 474 | | axSpA |
| Garrido-Cumbrera M, et al; EMAS working group. RMD Open, 2021 [50] | Multicenter,  cross-sectional | | 12 European countries | | 2166 | | axSpA |
| Gok K, et al. J Clin Rheumatol, 2018 [51] | Single-center,  cross-sectional | | Turkey | | 185 | | axSpA |
| Gossec L, et al. Joint Bone Spine, 2016 [52] | Multicenter, prospective, observational cohort | | France | | 486 | | axSpA |
| Gözüküçük M, et al. Clin Exp Rheumatol, 2022 [53] | Single-center, cross-sectional, case-control | | Turkey | | 98 (+ 99 healthy controls) | | axSpA |
| Günaydin R, et al. Clin Rheumatol, 2009 [54] | Single-center,  cross-sectional | | Turkey | | 62 | | AS |
| Hakkou J, et al. J Clin Rheumatol, 2011 [55] | Single-center,  cross-sectional | | Morocco | | 110 | | AS |
| Hakkou J, et al. Rheumatol Int, 2013 [56] | Single-center,  cross-sectional | | Morocco | | 110 | | AS |
| Haywood KL, et al. Rheumatology (Oxford), 2014 [57] | Multicenter,  cross-sectional | | UK | | 612 | | AS |
| Healey EL, et al. Scand J Rheumatol, 2011 [58] | Multicenter,  cross-sectional | | UK | | 612 | | AS |
| Healey EL, et al. Rheumatology (Oxford), 2009 [59] | Multicenter,  cross-sectional | | UK | | 612 | | AS |
| Heiberg T, et al. Ann Rheum Dis, 2011 [60] | Post hoc, exploratory analysis | | Norway | | 237 | | AS |
| Huang JC, et al. BMC Musculoskelet Disord, 2017 [61] | Retrospective  case series | | China | | 245 | | AS |
| Hwang MC, et al. Rheumatol Int, 2020 [62] | Multicenter, prospective, observational cohort | | USA | | 991 | | AS |
| Ibn Yacoub Y, et al. Clin Rheumatol, 2010 [63] | Single-center,  cross-sectional | | Morocco | | 100 | | AS |
| Ibn Yacoub Y, et al. Clin Rheumatol, 2011 [64] | Single-center,  cross-sectional | | Morocco | | 100 | | AS |
| Jiang Y, et al. Clin Rheumatol, 2018 [65] | Multicenter,  cross-sectional | | China | | 2772 (+ unknown no. of volunteers) | | AS |
| Jiang Y, et al. Clin Rheumatol, 2015 [66] | Multicenter,  cross-sectional | | China | | 683 | | AS |
| Kankaya H, et al. J Transcult Nurs, 2023 [67] | Single-center,  cross-sectional | | Turkey | | 101 | | AS |
| Karatekin BD, et al. J Coll Physicians Surg Pak, 2023 [68] | Single-center, prospective, observational cohort | | Turkey | | 41 | | AS |
| Kedra J, et al. J Rheumatol, 2022 [69] | Multicenter,  cross-sectional | | France | | 638 | | axSpA |
| Kieskamp SC, et al. Semin Arthritis Rheum, 2022 [70] | Prospective, observational cohort | | The Netherlands | | 182 | | axSpA |
| Kiltz U, et al. RMD Open, 2023 [71] | Multicenter,  cross-sectional | | Germany | | 770 | | axSpA |
| Korotaeva T, et al. Clin Rheumatol, 2021 [72] | Cross-sectional | | USA | | 487 | | AS |
|  |  |  | CEE | | 922 | |  |
| Kydd AS, et al. Rheumatology (Oxford), 2015 [73] | Retrospective, longitudinal cohort | | Australia | | 422 | | AS |
| Landewé R, et al. Ann Rheum Dis, 2009 [74] | Multicenter, prospective, observational cohort | | The Netherlands, Belgium, France | | 217 | | AS |
| Law L, et al. Arthritis Res Ther, 2018 [75] | Multicenter,  case-control | | Sweden | | 210 (+ 1055 healthy volunteers) | | AS |
| Li Y, et al. Arthritis Res Ther, 2012 [76] | Multicenter,  case-control | | China | | 314 (+ 102 healthy controls) | | AS |
| Lu MC, et al. BMJ Open, 2019 [77] | Single-center,  cross-sectional | | Taiwan | | 265 | | AS |
| Machado P, et al. Ann Rheum Dis, 2011 [78] | Post hoc, exploratory,  cross-sectional analysis | | Europe and USA | | 224 | | AS |
| Magrey M, et al. ACR Open Rheumatol, 2023 [79] | Multicenter,  cross-sectional | | USA | | 228 | | axSpA |
| Maguire S, et al. Scand J Rheumatol, 2022 [80] | Multicenter,  cross-sectional | | Ireland | | 759 | | axSpA |
| Maguire S, et al. J Rheumatol, 2022 [81] | Multicenter,  cross-sectional | | Ireland | | 753 | | axSpA |
| Maksymowych WP, et al. J Rheumatol, 2010 [82] | Multicenter, randomized,  double-blind,  placebo-controlled | | USA | | 315 | | AS |
| Mattey DL, et al. J Rheumatol, 2011 [83] | Multicenter,  cross-sectional | | UK | | 606 | | AS |
| Mease PJ, et al. J Rheumatol, 2021 [84] | Multicenter, prospective, observational cohort | | USA | | 498 | | axSpA |
| Mlcoch T, et al. Expert Rev Pharmacoecon Outcomes Res, 2017 [85] | Multicenter, prospective, observational cohort | | Czech Republic | | 313 | | AS |
| Morton L, et al. Arthritis Care Res (Hoboken), 2022 [86] | Multicenter,  cross-sectional | | Scotland | | 718 | | axSpA |
| Mustur D, et al. Srp Arh Celok Lek, 2009 [87] | Single-center,  cross-sectional | | Montenegro | | 74 | | AS |
| Nam B, et al. Qual Life Res, 2021 [88] | Single-center,  cross-sectional | | Republic of Korea | | 211 | | AS |
| Nie A, et al. Clin Rheumatol, 2018 [89] | Single-center,  cross-sectional | | China | | 281 | | AS |
| Nikiphorou E, et al. Rheumatology (Oxford), 2022 [90] | Multicenter, prospective, observational cohort | | France | | 704 | | Early axSpA |
| Nikiphorou E, et al. Arthritis Care Res (Hoboken), 2018 [91] | Multicenter,  cross-sectional | | Africa, Americas, Asia, Europe | | 3370 | | axSpA |
| O'Dwyer T, et al. Rheumatol Int, 2015 [92] | Single-center,  cross-sectional, controlled | | Ireland | | 39 (+ 39 controls) | | AS |
| O'Dwyer T, et al. Physiotherapy, 2016 [93] | Single-center,  cross-sectional, controlled | | Ireland | | 39 (+ 39 controls) | | AS |
| Öğüt TS, et al. Clin Rheumatol, 2023 [94] | Single-center,  cross-sectional | | Turkey | | 175 | | axSpA |
| Öksüz E, et al. Perspect Psychiatr Care, 2021 [95] | Single-center,  cross-sectional | | Turkey | | 119 | | AS |
| Omar M, et al. J Affect Disord, 2023 [96] | Retrospective, population-based, cross-sectional | | Israel | | 5825 (+ 25 984 controls) | | AS |
| Ortancil O, et al. J Clin Rheumatol, 2010 [97] | Single-center,  cross-sectional | | Turkey | | 29 (+ 20 controls) | | AS |
| Ozdemir O. Rheumatol Int, 2011 [98] | Single-center,  cross-sectional | | Turkey | | 48 | | AS |
| Park JS, et al. Sci Rep, 2019 [99] | Multicenter, prospective, observational cohort | | Republic of Korea | | 11 465 (+  57 325 controls) | | AS |
| Poddubnyy D, et al. Arthritis Rheum, 2012 [100] | Multicenter, prospective cohort | | Germany | | 210 | | axSpA |
| Poddubnyy D, et al. Ann Rheum Dis, 2013 [101] | Multicenter, prospective cohort | | Germany | | 210 | | axSpA |
| Rafia R, et al. Clin Exp Rheumatol, 2012 [102] | Multicenter,  cross-sectional | | UK | | 612 | | AS |
| Redeker I, et al. Ann Rheum Dis, 2018 [103] | Multicenter  cross-sectional | | Germany | | 1736 | | axSpA |
| Rezvani A, et al. Mod Rheumatol, 2014 [104] | Cross-sectional | | Turkey | | 421 | | AS |
| Rohde G, et al. Qual Life Res, 2020 [105] | Multicenter, prospective, observational cohort | | Norway | | 380 | | axSpA |
| Rosas J, et al; and the AIRE-MB Group. Clin Exp Rheumatol, 2017 [106] | Single-center,  cross-sectional | | Spain | | 57 | | AS |
| Rostom S, et al. Rheumatol Int, 2013 [107] | Cross-sectional | | Morocco | | 110 | | AS |
| Sağ S, et al. J Back Musculoskelet Rehabil, 2018 [108] | Single-center,  cross-sectional | | Turkey | | 50 (+ 30 controls) | | AS |
| Sakellariou GT, et al. Rheumatol Int, 2015 [109] | Single-center,  cross-sectional | | Greece | | 106 | | AS |
| Santana T, et al. Int Braz J Urol, 2017 [110] | Single-center,  cross-sectional | | Brazil | | 40 (+ 40 controls) | | AS |
| Sariyildiz MA, et al. Rheumatol Int, 2013 [111] | Multicenter,  cross-sectional | | Turkey | | 70 (+ 60 controls) | | AS |
| Sariyildiz MA, et al. Int J Impot Res, 2013 [112] | Multicenter,  cross-sectional | | Turkey | | 37 (+ 33 controls) | | AS |
| Schmalz G, et al. Clin Oral Investig, 2018 [113] | Single-center,  cross-sectional | | Germany | | 50 (+ 50 controls) | | AS |
| Schneeberger EE, et al. Clin Rheumatol, 2015 [114] | Case-control | | Argentina | | 64 (+ 95 controls) | | AS |
| Shahlaee A, et al. Clin Rheumatol, 2015 [115] | Single-center,  cross-sectional | | Iran | | 320 | | AS |
| Shen B, et al. Rheumatol Int, 2013 [116] | Single-center,  cross-sectional | | China | | 103 (+ 121 controls) | | AS |
| Shen B, et al. Psychol Psychother, 2014 [117] | Single-center,  cross-sectional | | China | | 103 (+ 121 controls) | | AS |
| Shen CC, et al. J Rheumatol, 2016 [118] | Multicenter, retrospective cohort | | Taiwan | | 2331 (+ 9324 controls) | | AS |
| Sieper J, et al. Clin Exp Rheumatol, 2016 [119] | Multicenter,  cross-sectional | | Europe (Germany, France, Spain, Italy, UK) | | 631 | | nr-axSpA |
| Slobodin G, et al. Clin Rheumatol, 2011 [120] | Multicenter,  cross-sectional | | Israel | | 151 | | Early axSpA |
| Stebbings SM, et al. Clin Rheumatol, 2014 [121] | Single-center,  cross-sectional | | New Zealand | | 67 | | axSpA |
| Strand V, et al. J Clin Rheumatol, 2021 [122] | Multinational,  cross-sectional | | North America, Europe, Asia Pacific, Middle East | | 705 | | AS |
| Swinnen TW, et al. Arthritis Res Ther, 2018 [123] | Single-center,  cross-sectional | | Belgium | | 170 | | axSpA |
| Tam LS, et al. J Rheumatol, 2007 [124] | Multicenter,  cross-sectional | | Hong Kong | | 314 | | AS |
| Tarhan F, et al. Int J Androl, 2012 [125] | Single-center,  cross-sectional | | Turkey | | 50 (+ 50 healthy controls) | | AS |
| Taser B, et al. Acta Reumatol Port, 2017 [126] | Single-center,  cross-sectional | | Turkey | | 100 | | AS |
| Tu L, et al. Clin Exp Rheumatol, 2014 [127] | Single-center,  cross-sectional | | China | | 257 | | AS |
| Turan Y, et al. Rheumatol Int, 2007 [128] | Single-center,  cross-sectional | | Turkey | | 68 | | AS |
| Turan Y, et al. Rheumatol Int, 2007 [129] | Cross-sectional | | Turkey | | 46 | | AS |
| Tymms K, et al. Clin Rheumatol, 2022 [130] | Multicenter,  cross-sectional | | Australia | | 495 | | AS |
| Uludag M, et al. Rheumatol Int, 2012 [131] | Single-center,  cross-sectional | | Turkey | | 25 (+ their spouses + 25 healthy controls) | | AS |
| Ulus Y, et al. Clin Rheumatol, 2019 [132] | Single-center,  cross-sectional, case-control | | Turkey | | 61 (+ 40 controls) | | AS |
| Urkmez B, Keskin Y. Mod Rheumatol, 2020 [133] | Cross-sectional | | Turkey | | 150 (+ 80 controls) | | AS |
| van der Heijde D, et al. Rheumatology (Oxford), 2016 [134] | Post hoc analysis of a multicenter, Phase 3 RCT | | Australia, Belgium,  Canada, Czech Republic, France, Germany, Spain, the Netherlands,  UK, USA | | 179 | | TNFi-IR  nr-axSpA |
| van der Horst-Bruinsma IE, et al. Ann Rheum Dis, 2013 [135] | Post hoc, exploratory analysis | | The Netherlands | | 1283 | | AS |
| van Genderen S, et al. Arthritis Care Res (Hoboken), 2016 [136] | Multicenter  cross-sectional | | The Netherlands | | 246 (+ 784 controls) | | AS |
| van Genderen S, et al. Arthritis Care Res (Hoboken), 2018 [137] | Multicenter  cross-sectional | | The Netherlands | | 246 (+ 510 controls) | | AS |
| van Genderen S, et al. J Clin Rheumatol, 2014 [138] | Case-control study | | The Netherlands | | 24 (+ 24 controls) | | AS |
| van Lunteren M, et al. J Rheumatol, 2018 [139] | Multinational, prospective cohort | | Europe | | 161 | | Early axSpA |
| van Lunteren M, et al. Rheumatology (Oxford), 2017 [140] | Multinational, prospective cohort | | Europe | | 105 | | Early axSpA |
| Vergne-Salle P, et al. Nutrients, 2022 [141] | Single-center, non-interventional cohort | | France | | 235 | | axSpA |
| Vesović-Potić V, et al. Rheumatol Int, 2009 [142] | Single-center,  cross-sectional | | Montenegro | | 74 | | AS |
| Vitturi BK, et al. Can J Neurol Sci, 2020 [143] | Single-center,  cross-sectional,  case-control | | Brazil | | 40 (+ 40 controls) | | AS |
| Wadeley A, et al. Clin Rheumatol, 2018 [144] | Single-center,  cross-sectional | | UK | | 659 | | axSpA |
| Walsh JA, et al. Clin Rheumatol, 2018 [145] | Retrospective, observational cohort | | USA | | 6679 (+ 19951 controls) | | AS |
| Ward MM, et al. Arthritis Rheum, 2008 [146] | Multicenter,  cross-sectional | | USA | | 591 | | AS |
| Wu JJ, et al. J Eur Acad Dermatol Venereol, 2017 [147] | Population-based cohort | | USA | | 1878 | | AS |
| Yılmaz O, et al. Rheumatol Int, 2013 [148] | Single-center, prospective, observational | | Turkey | | 74 | | AS |
| Yüce E, et al. Agri, 2023 [149] | Single-center,  cross-sectional, case-control | | Turkey | | 100 (+ 100 controls) | | AS |
| Žagar I, et al. Psychiatr Danub, 2021 [150] | Single-center,  cross-sectional | | Croatia | | 150 | | AS |
| Zhang H, et al. Clin Rheumatol, 2018 [151] | Multicenter,  cross-sectional | | China | | 1178 | | AS |
| Zhou W, et al. Clin Rheumatol, 2020 [152] | Single-center,  cross-sectional | | China | | 150 | | AS |
| Zviahina OV, et al. Wiad Lek, 2020 [153] | Single-center,  cross-sectional | | Ukraine | | 118 | | AS |
| B. Studies from journals not indexed^a^ in PubMed (*n* = 7) | | | | | | | |
| Hwang MC, et al. ACR Open Rheumatol, 2022 [154] | Retrospective cohort | | USA | | 7744 | | AS |
| Mease PJ, et al. ACR Open Rheumatol, 2020 [155] | Multicenter, prospective, observational | | USA | | 477 | | axSpA |
| Rausch Osthoff AK, et al. BMC Rheumatology, 2022 [156] | Cross-sectional | | Switzerland | | 24 | | axSpA |
| Park JYE, et al. BMC Rheumatology, 2020 [157] | Systematic review  and meta-analysis | | Global  (US, Taiwan, China, Turkey, Sweden) | | N/A | | AS |
| Phang JK, et al. BMC Rheumatology, 2021 [158] | Cross-sectional | | Singapore | | 74 | | axSpA |
| Tang H, et al. Rheumatol Adv Pract, 2023 [159] | Systematic review  and meta-analysis | | Global (Asia, America, UK, Europe, Africa) | | 7893 | | axSpA |
| Wilson N, et al. BMC Rheumatology, 2023 [160] | Systematic review and thematic synthesis of qualitative studies  and review of social media | | Global (UK, Europe, America, Asia) | | 1314 | | axSpA |

^a^At the time of the search.

AS: ankylosing spondylitis; axSpA: axial spondyloarthritis; CEE: Central Eastern Europe; IR: inadequate response; nr-axSpA: non-radiographic axSpA; TNFi: tumor necrosis factor inhibitor.

| Table S4. Conference proceedings (*N* = 37) | | | | | | | | |
| --- | --- | --- | --- | --- | --- | --- | --- | --- |
| Publication, year [Ref] | **Type** | **Location** | | **Conference** | **Number of patients** | | | **Disease** |
| Chang YS, et al. Int J Rheum Dis, 2018 [161] | Cross-sectional | Taiwan | APLAR | | | 88 | AS | |
| De Silva S, et al. Int J Rheum, 2021 [162] | Single-center, cross-sectional | Sri Lanka | APLAR | | | 35 | axSpA | |
| Dong C, et al. Int J Rheum Dis, 2018 [163] | Population-based, cross-sectional | China | APLAR | | | 81 | AS | |
| Ediboglu E, et al. Arthritis Rheumatol, 2020 [164] | Single-center, cross-sectional | Turkey | ACR | | | 219 | axSpA | |
| Fongen C, et al. Ann Rheum Dis, 2023 [165] | Multicenter, cross-sectional | Norway | EULAR | | | 99 | axSpA | |
| Garrido-Cumbrera M, et al. J Clin Rheumatol, 2021 [166] | Multinational, population-based, cross-sectional | Europe | PANLAR | | | 2846 | axSpA | |
| Garrido-Cumbrera M, et al. Ann Rheum Dis, 2020 [167] | Multinational, population-based, cross-sectional | Europe | EULAR | | | 2846 | axSpA | |
| Garrido-Cumbrera M, et al. Arthritis Rheumatol, 2020 [168] | Multicenter, cross-sectional | Spain | ACR | | | 680 | axSpA | |
| Garrido-Cumbrera M, et al. Ann Rheum Dis, 2018 [169] | Cross-sectional | Spain | EULAR | | | 680 | axSpA | |
| Garrido-Cumbrera M, et al. Ann Rheum Dis, 2021 [170] | Multicenter, cross-sectional study | Europe | EULAR | | | 2846 | axSpA | |
| Garrido-Cumbrera M, et al. Ann Rheum Dis, 2020 [171] | Multinational, population-based, cross-sectional | Europe | EULAR | | | 2846 | axSpA | |
| Garrido-Cumbrera M, et al. Arthritis Rheumatol, 2021 [172] | Multicenter, cross-sectional | Europe | ACR | | | 2846 | axSpA | |
| Garrido-Cumbrera M, et al. Ann Rheum Dis, 2020 [173] | Multicenter, cross-sectional | Europe | EULAR | | | 2846 | axSpA | |
| Navarro-Compán V, et al. J Clin Rheumatol, 2021 [174] | Multinational, population-based, cross-sectional | Europe | PANLAR | | | 2846 | axSpA | |
| Gossec L, et al. Arthritis Rheumatol, 2018 [175] | Multicenter, cross-sectional | Europe | ACR | | | 2846 | axSpA | |
| Ibanez S, et al. Arthritis Rheumatol, 2019 [176] | Population-based, cross-sectional | Chile | ACR | | | 472 | axSpA | |
| Kabir T, et al. Int J Rheum Dis, 2021 [177] | Single-center, cross-sectional | Bangladesh | APLAR | | | 76 | axSpA | |
| Kleinert S, et al. Arthritis Rheumatol, 2021 [178] | Double-center, cross-sectional | Germany | ACR | | | 101 | axSpA | |
| Kwan YH, et al, 2018 [179] | Prospective, observational | Singapore | SingHealth Duke-NUS Scientific Congress | | | 221 | axSpA | |
| Lim W, et al. Arthritis Rheumatol, 2020 [180] | Single-center, cross-sectional | Singapore | ACR | | | 262 | axSpA | |
| Liu LC, et al. Int J Rheum Dis, 2018 [181] | Single-center, cross-sectional | Taiwan | APLAR | | | 265 | AS | |
| Liu X, et al. Arthritis Rheumatol, 2019 [182] | Population-based, cross-sectional | China | ACR | | | 1187 | axSpA | |
| Lopez-Medina C, et al. Arthritis Rheumatol, 2022 [183] | Multicenter, cross-sectional | Spain | ACR | | | 866 | axSpA | |
| Maguire S, et al. Arthritis Rheumatol, 2021 [184] | Population-based, cross-sectional | Ireland | ACR | | | 876 | axSpA | |
| Maguire S, et al. Arthritis Rheumatol, 2021 [185] | Population-based, cross-sectional | Ireland | ACR | | | 220 | axSpA | |
| Maguire S, et al. Arthritis Rheumatol, 2021 [186] | Single-center, cross-sectional | Ireland | ACR | | | 71 | axSpA | |
| Poddubnyy D, et al. Arthritis Rheumatol, 2021 [187] | Real-world, prospective, observational | Multinational | ACR | | | 2633 | axSpA | |
| Polanco Mora T, et al. J Clin Rheumatol, 2021 [188] | Single-center, cross-sectional | Dominican Republic | PANLAR | | | 88 | AS | |
| Redeker I, et al. Ann Rheum Dis, 2018 [189] | Multicenter, cross-sectional | Germany | EULAR | | | 1736 | axSpA | |
| Regierer AC, et al. Ann Rheum Dis, 2020 [190] | Prospective, longitudinal cohort | Germany | EULAR | | | 848 | axSpA | |
| Rondags A, et al. Ann Rheum Dis, 2018 [191] | Cross-sectional | The Netherlands | EULAR | | | 449 | axSpA | |
| Rosenbaum JT, et al. Arthritis Rheumatol, 2018 [192] | Real-world, cross-sectional | USA | ACR | | | 716 | AS | |
| Sagard J, et al. Arthritis Rheumatol, 2020 [193] | Population-based, cross-sectional | Sweden | ACR | | | 254  (nr‑axSpA: 86  AS: 168) | axSpA | |
| Samreen S, et al. Int J Rheum, 2022 [194] | Single-center, cross-sectional | Pakistan | APLAR | | | 100 | axSpA | |
| Telugu S, et al. Int J Rheum Dis, 2021 [195] | Cross-sectional | India | APLAR | | | 38 | axSpA | |
| Ursin K, et al. Ann Rheum Dis, 2018 [196] | Population-based, cross-sectional | Norway | EULAR | | | 166  (179 pregnancies) | axSpA | |
| van der Meer R, et al. Ann Rheum Dis, 2018 [197] | Prospective cohort | The Netherlands | EULAR | | | 421 | axSpA | |

ACR: American College of Rheumatology; APLAR: Asia Pacific League of Associations for Rheumatology; AS: ankylosing spondylitis; axSpA: axial spondyloarthritis; EULAR: European Alliance of Associations for Rheumatology; nr-axSpA: non-radiographic axSpA; PANLAR: Pan-American League of Associations for Rheumatology.

| Table S5. Articles identified in the literature investigating disease activity in association with psychosocial outcomes (*N* = 138) | | | | | | |
| --- | --- | --- | --- | --- | --- | --- |
| Publication, year [Ref] | **Study type** | **Location** | | **Number of patients** | **Disease** | |
| Aggarwal R, Malaviya AN. Clin Rheumatol, 2009 [1] | Single-center, prospective cohort | | India | 70 | | AS |
| Aissaoui N, et al. Rheumatol Int, 2012 [2] | Cross-sectional | | Morocco | 110 | | AS |
| Alkan BM, et al. Mod Rheumatol, 2013 [3] | Cross-sectional | | Turkey | 110 (+ 40 healthy volunteers) | | AS |
| Ariza-Ariza R, et al. J Rheumatol, 2009 [4] | Cross-sectional | | Spain | 699 | | AS |
| Atar E, Askin A. Int J Rheum Dis, 2020 [5] | Cross-sectional | | Turkey | 80 | | AS |
| Aykurt Karlıbel İ, et al. Aging Male, 2019 [6] | Prospective observational cohort | | Turkey | 67 | | AS |
| Aykurt Karlıbel I, Kasapoğlu Aksoy M. Ir J Med Sci, 2023 [7] | Cross-sectional | | Turkey | 82 (+ 40 healthy volunteers) | | axSpA |
| Bal S, et al. Rheumatol Int, 2011 [9] | Single-center, prospective, observational | | Turkey | 37 (+ 67 healthy controls) | | AS |
| Bandinelli F, et al. Clin Rheumatol, 2016 [10] | Single-center, retrospective cohort | | Italy | 135 | | AS |
| Batmaz İ, et al. Rheumatol Int, 2013 [11] | Single-center,  cross-sectional | | Turkey | 80 (+ 52 controls) | | AS |
| Baysal O, et al. Rheumatol Int, 2011 [12] | Multicenter, cross-sectional | | Turkey | 243 (+ 118 healthy controls) | | AS |
| Bedaiwi M, et al. J Rheumatol, 2015 [13] | Longitudinal cohort | | Canada | 681 | | axSpA |
| Bodur H, et al. Qual Life Res, 2011 [14] | Single-center,  cross-sectional | | Turkey | 962 | | AS |
| Boonen A, et al. J Rheumatol, 2015 [15] | Prospective, multicenter, open-label, observational cohort | | Belgium | 80 | | AS |
| Boonen A, et al. Rheumatology (Oxford), 2018 [16] | Prospective, multicenter, open-label, observational cohort | | Belgium | 75 | | AS |
| Brophy S, et al. Semin Arthritis Rheum, 2013 [17] | Prospective, multicenter, observational | | UK | 385 | | AS |
| Cakar E, et al. Clin Rheumatol, 2007 [18] | Double-center, cross-sectional | | Turkey | 53 | | AS |
| Cakar E, et al. Clin Rheumatol, 2009 [19] | Prospective, observational | | Turkey | 121 | | AS |
| Carvalho PD, et al. Arthritis Care Res (Hoboken), 2022 [20] | Prospective, multicenter, observational cohort | | France | 644 | | Early axSpA |
| Cengiz G, et al. Int J Rheum Dis, 2023 [21] | Single-center,  cross-sectional | | Turkey | 110 | | axSpA |
| Chen CH, et al. Int J Rheum Dis, 2020 [22] | Single-center,  cross-sectional | | Taiwan | 105 | | AS |
| Chen HH, et al. PLoS One, 2020 [24] | Single-center,  cross-sectional | | Taiwan | 307 | | AS |
| Chen MH, et al. Clin Rheumatol, 2018 [25] | Multicenter, retrospective | | Taiwan | 120 | | AS |
| Chen CH, et al. Clin Rheumatol, 2013 [23] | Single-center,  cross-sectional | | Taiwan | 75 | | AS |
| Chung DXY, et al. BMJ Open, 2023 [26] | Single-center,  cross-sectional | | Singapore | 296 | | axSpA |
| Chung HY, et al. Ann Rheum Dis, 2012 [27] | Multicenter, cross-sectional | | France | 647 | | axSpA |
| Connolly D, et al. Occup Ther Int, 2019 [28] | Single-center,  cross-sectional | | Ireland | 50 | | AS |
| Cunha RN, et al. ARP Rheumatol, 2022 [29] | Multicenter, cross-sectional | | Portugal | 1995 | | axSpA |
| D'Angelo S, et al. Clin Exp Rheumatol, 2019 [30] | Multicenter, prospective, observational | | Italy | 512 | | axSpA |
| D'Angelo S, et al. Clin Exp Rheumatol, 2021 [31] | Multicenter, prospective, observational | | Italy | 512 | | axSpA |
| Demir SE, et al. Rheumatol Int, 2013 [32] | Single-center,  cross-sectional | | Turkey | 23 (+ 27 healthy controls) | | AS |
| Dhakad U, et al. Int J Rheum Dis, 2015 [33] | Single-center, prospective, case-control study | | India | 100 (+ 100 healthy controls) | | AS |
| Dincer U, et al. Tohoku J Exp Med, 2007 [34] | Single-center,  cross-sectional | | Turkey | 36 (+ 34 controls) | | AS |
| Dincer U, et al. Rheumatol Int, 2007 [35] | Single-center,  cross-sectional | | Turkey | 65 (+ 45 healthy controls) | | AS |
| Dülger S, et al. J Clin Rheumatol, 2019 [36] | Single-blind, controlled, observational | | Turkey | 92 | | AS |
| Durcan L, et al. J Rheumatol, 2012 [37] | Single-center,  cross-sectional | | Ireland | 46 | | AS |
| Durmus D, et al. Compr Psychiatry, 2015 [38] | Single-center,  cross-sectional | | Turkey | 80 (+ 80 healthy controls) | | AS |
| Elolemy G, et al. Curr Rheumatol Rev, 2020 [39] | Multicenter, cross-sectional | | Kuwait and Saudi Arabia | 47 | | AS |
| Espahbodi S, et al. Clin Exp Rheumatol, 2017 [40] | Single-center,  cross-sectional | | UK | 490 | | axSpA |
| Essers I, et al. Rheumatology (Oxford), 2016 [41] | Multicenter, prospective cohort | | The Netherlands | 90 | | AS |
| Fernández-Carballido C, et al. Arthritis Care Res (Hoboken), 2020 [42] | Multicenter,  cross-sectional | | Spain | 738  (+ 721 with PsA) | | AS |
| Fernández-Carballido C, et al; Esperanza Study Group. Arthritis Care Res (Hoboken), 2017 [43] | Multicenter, cross-sectional | | Spain | 259 | | Early axSpA |
| Fongen C, et al. Musculoskeletal Care, 2015 [44] | Single-center,  cross-sectional | | Norway | 148 (+ 133 controls) | | AS |
| Forejtová S, et al. Clin Rheumatol, 2008 [45] | Single-center,  cross-sectional | | Czech Republic | 1001 | | AS |
| Frede N, et al. RMD Open, 2023 [46] | Single-center, retrospective, cross-sectional | | Austria | 162  (+ 168 with PsA) | | axSpA |
| Garrido-Cumbrera M, et al. Arthritis Care Res (Hoboken), 2021 [47] | Multicenter, cross-sectional | | 13 European countries | 2695 | | axSpA |
| Garrido-Cumbrera M, et al. J Rheumatol, 2022 [48] | Cross-sectional | | Spain | 653 | | axSpA |
| Garrido-Cumbrera M. J Rheumatol, 2019 [49] | Cross-sectional | | Spain | 474 | | axSpA |
| Garrido-Cumbrera M, et al; EMAS working group. RMD Open, 2021 [50] | Multicenter, cross-sectional | | 12 European countries | 2166 | | axSpA |
| Gok K, et al. J Clin Rheumatol, 2018 [51] | Single-center,  cross-sectional | | Turkey | 185 | | axSpA |
| Gossec L, et al. Joint Bone Spine, 2016 [52] | Multicenter, prospective, observational cohort | | France | 486 | | axSpA |
| Gözüküçük M, et al. Clin Exp Rheumatol, 2022 [53] | Single-center, cross-sectional, case-control | | Turkey | 98 (+ 99 healthy controls) | | axSpA |
| Günaydin R, et al. Clin Rheumatol, 2009 [54] | Single-center,  cross-sectional | | Turkey | 62 | | AS |
| Hakkou J, et al. J Clin Rheumatol, 2011 [55] | Single-center,  cross-sectional | | Morocco | 110 | | AS |
| Hakkou J, et al. Rheumatol Int, 2013 [56] | Single-center,  cross-sectional | | Morocco | 110 | | AS |
| Haywood KL, et al. Rheumatology (Oxford), 2014 [57] | Multicenter, cross-sectional | | UK | 612 | | AS |
| Healey EL, et al. Scand J Rheumatol, 2011 [58] | Multicenter, cross-sectional | | UK | 612 | | AS |
| Healey EL, et al. Rheumatology (Oxford), 2009 [59] | Multicenter, cross-sectional | | UK | 612 | | AS |
| Huang JC, et al. BMC Musculoskelet Disord, 2017 [61] | Retrospective case series | | China | 245 | | AS |
| Hwang MC, et al. Rheumatol Int, 2020 [62] | Multicenter, prospective, observational cohort | | USA | 991 | | AS |
| Ibn Yacoub Y, et al. Clin Rheumatol, 2010 [63] | Single-center,  cross-sectional | | Morocco | 100 | | AS |
| Ibn Yacoub Y, et al. Clin Rheumatol, 2011 [64] | Single-center,  cross-sectional | | Morocco | 100 | | AS |
| Jiang Y, et al. Clin Rheumatol, 2018 [65] | Multicenter, cross-sectional | | China | 2772 (+ unknown no. of volunteers) | | AS |
| Jiang Y, et al. Clin Rheumatol, 2015 [66] | Multicenter, cross-sectional | | China | 683 | | AS |
| Karatekin BD, et al. J Coll Physicians Surg Pak, 2023 [68] | Single-center, prospective, observational cohort | | Turkey | 41 | | AS |
| Kedra J, et al. J Rheumatol, 2022 [69] | Multicenter, cross-sectional | | France | 638 | | axSpA |
| Kieskamp SC, et al. Semin Arthritis Rheum, 2022 [70] | Prospective, observational cohort | | The Netherlands | 182 | | axSpA |
| Kiltz U, et al. RMD Open, 2023 [71] | Multicenter, cross-sectional | | Germany | 770 | | axSpA |
| Korotaeva T, et al. Clin Rheumatol, 2021 [72] | Cross-sectional | | USA | 487 | | AS |
|  |  |  | CEE | 922 | |  |
| Kydd AS, et al. Rheumatology (Oxford), 2015 [73] | Retrospective, longitudinal cohort | | Australia | 422 | | AS |
| Landewé R, et al. Ann Rheum Dis, 2009 [74] | Multicenter, prospective, observational cohort | | The Netherlands, Belgium, France | 217 | | AS |
| Law L, et al. Arthritis Res Ther, 2018 [75] | Multicenter, case-control | | Sweden | 210 (+ 1055 healthy volunteers) | | AS |
| Li Y, et al. Arthritis Res Ther, 2012 [76] | Multicenter, case-control | | China | 314 (+ 102 healthy controls) | | AS |
| Lu MC, et al. BMJ Open, 2019 [77] | Single-center,  cross-sectional | | Taiwan | 265 | | AS |
| Machado P, et al. Ann Rheum Dis, 2011 [78] | Post hoc, exploratory,  cross-sectional analysis | | Europe and USA | 224 | | AS |
| Magrey M, et al. ACR Open Rheumatol, 2023 [79] | Multicenter, cross-sectional | | USA | 228 | | axSpA |
| Maguire S, et al. Scand J Rheumatol, 2022 [80] | Multicenter, cross-sectional | | Ireland | 759 | | axSpA |
| Maguire S, et al. J Rheumatol, 2022 [81] | Multicenter,  cross-sectional | | Ireland | 753 | | axSpA |
| Maksymowych WP, et al. J Rheumatol, 2010 [82] | Multicenter, randomized, double-blind,  placebo-controlled | | USA | 315 | | AS |
| Mattey DL, et al. J Rheumatol, 2011 [83] | Multicenter, cross-sectional | | UK | 606 | | AS |
| Mease PJ, et al. J Rheumatol, 2021 [84] | Multicenter, prospective, observational cohort | | USA | 498 | | axSpA |
| Mease PJ, et al. ACR Open Rheumatol, 2020 [155] | Multicenter, prospective, observational | | USA | 477 | | axSpA |
| Mlcoch T, et al. Expert Rev Pharmacoecon Outcomes Res, 2017 [85] | Multicenter, prospective, observational cohort | | Czech Republic | 313 | | AS |
| Morton L, et al. Arthritis Care Res (Hoboken), 2022 [86] | Multicenter, cross-sectional | | Scotland | 718 | | axSpA |
| Nam B, et al. Qual Life Res, 2021 [88] | Single-center,  cross-sectional | | Republic of Korea | 211 | | AS |
| Nie A, et al. Clin Rheumatol, 2018 [89] | Single-center,  cross-sectional | | China | 281 | | AS |
| Nikiphorou E, et al. Rheumatology (Oxford), 2022 [90] | Multicenter, prospective, observational cohort | | France | 704 | | Early axSpA |
| Nikiphorou E, et al. Arthritis Care Res (Hoboken), 2018 [91] | Multicenter, cross-sectional | | Africa, Americas, Asia, Europe | 3370 | | axSpA |
| O'Dwyer T, et al. Rheumatol Int, 2015 [92] | Single-center,  cross-sectional, controlled | | Ireland | 39 (+ 39 controls) | | AS |
| O'Dwyer T, et al. Physiotherapy, 2016 [93] | Single-center,  cross-sectional, controlled | | Ireland | 39 (+ 39 controls) | | AS |
| Öğüt TS, et al. Clin Rheumatol, 2023 [94] | Single-center,  cross-sectional | | Turkey | 175 | | axSpA |
| Öksüz E, et al. Perspect Psychiatr Care, 2021 [95] | Single-center,  cross-sectional | | Turkey | 119 | | AS |
| Ortancil O, et al. J Clin Rheumatol, 2010 [97] | Single-center,  cross-sectional | | Turkey | 29 (+ 20 controls) | | AS |
| Ozdemir O. Rheumatol Int, 2011 [98] | Single-center,  cross-sectional | | Turkey | 48 | | AS |
| Poddubnyy D, et al. Ann Rheum Dis, 2013 [101] | Multicenter, prospective cohort | | Germany | 210 | | axSpA |
| Rafia R, et al. Clin Exp Rheumatol, 2012 [102] | Multicenter, cross-sectional | | UK | 612 | | AS |
| Redeker I, et al. Ann Rheum Dis, 2018 [103] | Multicenter, cross-sectional | | Germany | 1736 | | axSpA |
| Rezvani A, et al. Mod Rheumatol, 2014 [104] | Cross-sectional | | Turkey | 421 | | AS |
| Rohde G, et al. Qual Life Res, 2020 [105] | Multicenter, prospective, observational cohort | | Norway | 380 | | axSpA |
| Rosas J, et al; and the AIRE-MB Group. Clin Exp Rheumatol, 2017 [106] | Single-center,  cross-sectional | | Spain | 57 | | AS |
| Rostom S, et al. Rheumatol Int, 2013 [107] | Cross-sectional | | Morocco | 110 | | AS |
| Sağ S, et al. J Back Musculoskelet Rehabil, 2018 [108] | Single-center,  cross-sectional | | Turkey | 50 (+ 30 controls) | | AS |
| Sakellariou GT, et al. Rheumatol Int, 2015 [109] | Single-center,  cross-sectional | | Greece | 106 | | AS |
| Santana T, et al. Int Braz J Urol, 2017 [110] | Single-center,  cross-sectional | | Brazil | 40 (+ 40 controls) | | AS |
| Sariyildiz MA, et al. Rheumatol Int, 2013 [111] | Multicenter, cross-sectional | | Turkey | 70 (+ 60 controls) | | AS |
| Sariyildiz MA, et al. Int J Impot Res, 2013 [112] | Multicenter, cross-sectional | | Turkey | 37 (+ 33 controls) | | AS |
| Schmalz G, et al. Clin Oral Investig, 2018 [113] | Single-center,  cross-sectional | | Germany | 50 (+ 50 controls) | | AS |
| Schneeberger EE, et al. Clin Rheumatol, 2015 [114] | Case-control | | Argentina | 64 (+ 95 controls) | | AS |
| Shahlaee A, et al. Clin Rheumatol, 2015 [115] | Single-center,  cross-sectional | | Iran | 320 | | AS |
| Shen B, et al. Rheumatol Int, 2013 [116] | Single-center,  cross-sectional | | China | 103 (+ 121 controls) | | AS |
| Shen B, et al. Psychol Psychother, 2014 [117] | Single-center,  cross-sectional | | China | 103 (+ 121 controls) | | AS |
| Stebbings SM, et al. Clin Rheumatol, 2014 [121] | Single-center, cross-sectional | | New Zealand | 67 | | axSpA |
| Strand V, et al. J Clin Rheumatol, 2021 [122] | Multinational,  cross-sectional | | North America, Europe, Asia Pacific, Middle East | 705 | | AS |
| Swinnen TW, et al. Arthritis Res Ther, 2018 [123] | Single-center,  cross-sectional | | Belgium | 170 | | axSpA |
| Tam LS, et al. J Rheumatol, 2007 [124] | Multicenter, cross-sectional | | Hong Kong | 314 | | AS |
| Taser B, et al. Acta Reumatol Port, 2017 [126] | Single-center,  cross-sectional | | Turkey | 100 | | AS |
| Tu L, et al. Clin Exp Rheumatol, 2014 [127] | Single-center,  cross-sectional | | China | 257 | | AS |
| Turan Y, et al. Rheumatol Int, 2007 [128] | Single-center,  cross-sectional | | Turkey | 68 | | AS |
| Turan Y, et al. Rheumatol Int, 2007 [129] | Cross-sectional | | Turkey | 46 | | AS |
| Tymms K, et al. Clin Rheumatol, 2022 [130] | Multicenter, cross-sectional | | Australia | 495 | | AS |
| Uludag M, et al. Rheumatol Int, 2012 [131] | Single-center,  cross-sectional | | Turkey | 25 (+ their spouses + 25 healthy controls) | | AS |
| Ulus Y, et al. Clin Rheumatol, 2019 [132] | Single-center,  cross-sectional  case-control | | Turkey | 61 (+ 40 controls) | | AS |
| Urkmez B, Keskin Y. Mod Rheumatol, 2020 [133] | Cross-sectional | | Turkey | 150 (+ 80 controls) | | AS |
| van der Heijde D, et al. Rheumatology (Oxford), 2016 [134] | Post hoc analysis of a  multicenter, Phase 3 RCT | | Australia, Belgium,  Canada, Czech Republic, France, Germany, Spain, the Netherlands,  UK, USA | 179 | | TNFi-IR  nr-axSpA |
| van der Horst-Bruinsma IE, et al. Ann Rheum Dis, 2013 [135] | Post hoc, exploratory analysis | | The Netherlands | 1283 | | AS |
| van Genderen S, et al. J Clin Rheumatol, 2014 [138] | Case-control study | | The Netherlands | 24 (+ 24 controls) | | AS |
| van Lunteren M, et al. J Rheumatol, 2018 [139] | Multinational, prospective cohort | | Europe | 161 | | Early axSpA |
| van Lunteren M, et al. Rheumatology (Oxford), 2017 [140] | Multinational, prospective cohort | | Europe | 105 | | Early axSpA |
| Vergne-Salle P, et al. Nutrients, 2022 [141] | Single-center, non-interventional cohort | | France | 235 | | axSpA |
| Vesović-Potić V, et al. Rheumatol Int, 2009 [142] | Single-center, cross-sectional | | Montenegro | 74 | | AS |
| Vitturi BK, et al. Can J Neurol Sci, 2020 [143] | Single-center,  cross-sectional,  case-control | | Brazil | 40 (+ 40 controls) | | AS |
| Wadeley A, et al. Clin Rheumatol, 2018 [144] | Single-center, cross-sectional | | UK | 659 | | axSpA |
| Yılmaz O, et al. Rheumatol Int, 2013 [148] | Single-center, prospective, observational | | Turkey | 74 | | AS |
| Yüce E, et al. Agri, 2023 [149] | Single-center, cross-sectional,  case-control | | Turkey | 100 (+ 100 controls) | | AS |
| Žagar I, et al. Psychiatr Danub, 2021 [150] | Single-center, cross-sectional | | Croatia | 150 | | AS |
| Zhang H, et al. Clin Rheumatol, 2018 [151] | Multicenter, cross-sectional | | China | 1178 | | AS |
| Zhou W, et al. Clin Rheumatol, 2020 [152] | Single-center, cross-sectional | | China | 150 | | AS |
| Zviahina OV, et al. Wiad Lek, 2020 [153] | Single-center, cross-sectional | | Ukraine | 118 | | AS |

AS: ankylosing spondylitis; axSpA: axial spondyloarthritis; nr-axSpA: non-radiographic axSpA; PsA: psoriatic arthritis; TNFi-IR: inadequate response to tumor necrosis factor inhibitor.

| Table S6. Articles identified in the literature investigating pain in association with psychosocial outcomes (*N* = 43) | | | | | | | |
| --- | --- | --- | --- | --- | --- | --- | --- |
| Publication, year [Ref] | **Study type** | **Location** | | **Number of patients** | | **Disease** | |
| Aissaoui N, et al. Rheumatol Int, 2012 [2] | Cross-sectional | | Morocco | | 110 | | AS |
| Alkan BM, et al. Mod Rheumatol, 2013 [3] | Cross-sectional | | Turkey | | 110 (+ 40 healthy volunteers) | | AS |
| Atar E, Askin A. Int J Rheum Dis, 2020 [5] | Cross-sectional | | Turkey | | 80 | | AS |
| Aykurt Karlıbel İ, et al. Aging Male, 2019 [6] | Prospective, observational cohort | | Turkey | | 67 | | AS |
| Aykurt Karlıbel I, Kasapoğlu Aksoy M. Ir J Med Sci, 2023 [7] | Cross-sectional | | Turkey | | 82 (+ 40 healthy volunteers) | | axSpA |
| Bal S, et al. Rheumatol Int, 2011 [9] | Single-center, prospective, observational | | Turkey | | 37 (+ 67 healthy controls) | | AS |
| Bandinelli F, et al. Clin Rheumatol, 2016 [10] | Single-center, retrospective cohort | | Italy | | 135 | | AS |
| Baysal O, et al. Rheumatol Int, 2011 [12] | Multicenter, cross-sectional | | Turkey | | 243 (+ 118 healthy controls) | | AS |
| Bodur H, et al. Qual Life Res, 2011 [14] | Single-center,  cross-sectional | | Turkey | | 962 | | AS |
| Brophy S, et al. Semin Arthritis Rheum, 2013 [17] | Prospective, multicenter, observational | | UK | | 385 | | AS |
| Cengiz G, et al. Int J Rheum Dis, 2023 [21] | Single-center,  cross-sectional | | Turkey | | 110 | | axSpA |
| Connolly D, et al. Occup Ther Int, 2019 [28] | Single-center,  cross-sectional | | Ireland | | 50 | | AS |
| D'Angelo S, et al. Clin Exp Rheumatol, 2021 [31] | Multicenter, prospective, observational | | Italy | | 512 | | axSpA |
| Demir SE, et al. Rheumatol Int, 2013 [32] | Single-center,  cross-sectional | | Turkey | | 23 (+ 27 healthy controls) | | AS |
| Durcan L, et al. J Rheumatol, 2012 [37] | Single-center,  cross-sectional | | Ireland | | 46 | | AS |
| Durmus D, et al. Compr Psychiatry, 2015 [38] | Single-center,  cross-sectional | | Turkey | | 80 (+ 80 healthy controls) | | AS |
| Elolemy G, et al. Curr Rheumatol Rev, 2020 [39] | Multicenter, cross-sectional | | Kuwait and Saudi Arabia | | 47 | | AS |
| Espahbodi S, et al. Clin Exp Rheumatol, 2017 [40] | Single-center,  cross-sectional | | UK | | 490 | | axSpA |
| Essers I, et al. Rheumatology (Oxford), 2016 [41] | Multicenter, prospective cohort | | The Netherlands | | 90 | | AS |
| Fongen C, et al. Musculoskeletal Care, 2015 [44] | Single-center,  cross-sectional | | Norway | | 148 (+ 133 controls) | | AS |
| Gok K, et al. J Clin Rheumatol, 2018 [51] | Single-center,  cross-sectional | | Turkey | | 185 | | axSpA |
| Gözüküçük M, et al. Clin Exp Rheumatol, 2022 [53] | Single-center, cross-sectional, case-control | | Turkey | | 98 (+ 99 healthy controls) | | axSpA |
| Healey EL, et al. Scand J Rheumatol, 2011 [58] | Multicenter, cross-sectional | | UK | | 612 | | AS |
| Healey EL, et al. Rheumatology (Oxford), 2009 [59] | Multicenter, cross-sectional | | UK | | 612 | | AS |
| Heiberg T, et al. Ann Rheum Dis, 2011 [60] | Post hoc, exploratory analysis | | Norway | | 237 | | AS |
| Ibn Yacoub Y, et al. Clin Rheumatol, 2011 [64] | Single-center,  cross-sectional | | Morocco | | 100 | | AS |
| Jiang Y, et al. Clin Rheumatol, 2015 [66] | Multicenter, cross-sectional | | China | | 683 | | AS |
| Li Y, et al. Arthritis Res Ther, 2012 [76] | Multicenter, case-control | | China | | 314 (+ 102 healthy controls) | | AS |
| Maksymowych WP, et al. J Rheumatol, 2010 [82] | Multicenter, randomized, double-blind,  placebo-controlled | | USA | | 315 | | AS |
| Mattey DL, et al. J Rheumatol, 2011 [83] | Multicenter, cross-sectional | | UK | | 606 | | AS |
| Mease PJ, et al. J Rheumatol, 2021 [84] | Multicenter, prospective, observational cohort | | USA | | 498 | | axSpA |
| Morton L, et al. Arthritis Care Res (Hoboken), 2022 [86] | Multicenter, cross-sectional | | Scotland | | 718 | | axSpA |
| Mustur D, et al. Srp Arh Celok Lek, 2009 [87] | Single-center,  cross-sectional | | Montenegro | | 74 | | AS |
| Öğüt TS, et al. Clin Rheumatol, 2023 [94] | Single-center, cross-sectional | | Turkey | | 175 | | axSpA |
| Ozdemir O. Rheumatol Int, 2011 [98] | Single-center, cross-sectional | | Turkey | | 48 | | AS |
| Shahlaee A, et al. Clin Rheumatol, 2015 [115] | Single-center, cross-sectional | | Iran | | 320 | | AS |
| Sieper J, et al. Clin Exp Rheumatol, 2016 [119] | Multicenter, cross-sectional | | Europe (Germany, France, Spain, Italy, UK) | | 631 | | nr-axSpA |
| Slobodin G, et al. Clin Rheumatol, 2011 [120] | Multicenter, cross-sectional | | Israel | | 151 | | Early axSpA |
| Strand V, et al. J Clin Rheumatol, 2021 [122] | Multinational, cross-sectional | | North America, Europe, Asia Pacific, Middle East | | 705 | | AS |
| Swinnen TW, et al. Arthritis Res Ther, 2018 [123] | Single-center, cross-sectional | | Belgium | | 170 | | axSpA |
| van der Horst-Bruinsma IE, et al. Ann Rheum Dis, 2013 [135] | Post hoc, exploratory analysis | | The Netherlands | | 1283 | | AS |
| Vesović-Potić V, et al. Rheumatol Int, 2009 [142] | Single-center, cross-sectional | | Montenegro | | 74 | | AS |
| Yılmaz O, et al. Rheumatol Int, 2013 [148] | Single-center, prospective, observational | | Turkey | | 74 | | AS |

AS: ankylosing spondylitis; axSpA: axial spondyloarthritis; nr-axSpA: non-radiographic axSpA.

| Table S7. Articles identified in the literature investigating morning stiffness in association with psychosocial outcomes (*N* = 10) | | | | | | |
| --- | --- | --- | --- | --- | --- | --- |
| Publication, year [Ref] | **Study type** | | **Location** | **Number of patients** | | **Disease** |
| Alkan BM, et al. Mod Rheumatol, 2013 [3] | Cross-sectional | Turkey | | | 110 (+ 40 healthy volunteers) | AS |
| Aykurt Karlıbel I, Kasapoğlu Aksoy M. Ir J Med Sci, 2023 [7] | Cross-sectional | Turkey | | | 82 (+ 40 healthy volunteers) | axSpA |
| Baysal O, et al. Rheumatol Int, 2011 [12] | Multicenter, cross-sectional | Turkey | | | 243 (+ 118 healthy controls) | AS |
| Dincer U, et al. Rheumatol Int, 2007 [35] | Single-center, cross-sectional | Turkey | | | 65 (+ 45 healthy controls) | AS |
| Heiberg T, et al. Ann Rheum Dis, 2011 [60] | Post hoc, exploratory analysis | Norway | | | 237 | AS |
| Ibn Yacoub Y, et al. Clin Rheumatol, 2011 [64] | Single-center, cross-sectional | Morocco | | | 100 | AS |
| Li Y, et al. Arthritis Res Ther, 2012 [76] | Multicenter, case-control | China | | | 314 (+ 102 healthy controls) | AS |
| Sariyildiz MA, et al. Int J Impot Res, 2013 [112] | Multicenter, cross-sectional | Turkey | | | 37 (+ 33 controls) | AS |
| Vesović-Potić V, et al. Rheumatol Int, 2009 [142] | Single-center, cross-sectional | Montenegro | | | 74 | AS |
| Yılmaz O, et al. Rheumatol Int, 2013 [148] | Single-center, prospective, observational | Turkey | | | 74 | AS |

AS: ankylosing spondylitis; axSpA: axial spondyloarthritis.

| Table S8. Articles identified in the literature investigating fatigue in association with psychosocial outcomes (*N* = 34) | | | | | | |
| --- | --- | --- | --- | --- | --- | --- |
| Publication, year [Ref] | **Study type** | | **Location** | **Number of patients** | | **Disease** |
| Aissaoui N, et al. Rheumatol Int, 2012 [2] | Cross-sectional | Morocco | | | 110 | AS |
| Alkan BM, et al. Mod Rheumatol, 2013 [3] | Cross-sectional | Turkey | | | 110 (+ 40 healthy volunteers) | AS |
| Aykurt Karlıbel İ, et al. Aging Male, 2019 [6] | Prospective, observational cohort | Turkey | | | 67 | AS |
| Aykurt Karlıbel I, Kasapoğlu Aksoy M. Ir J Med Sci, 2023 [7] | Cross-sectional | Turkey | | | 82 (+ 40 healthy volunteers) | axSpA |
| Bedaiwi M, et al. J Rheumatol, 2015 [13] | Longitudinal cohort | Canada | | | 681 | axSpA |
| Bodur H, et al. Qual Life Res, 2011 [14] | Single-center, cross-sectional | Turkey | | | 962 | AS |
| Brophy S, et al. Semin Arthritis Rheum, 2013 [17] | Prospective, multicenter, observational | UK | | | 385 | AS |
| Connolly D, et al. Occup Ther Int, 2019 [28] | Single-center, cross-sectional | Ireland | | | 50 | AS |
| Durmus D, et al. Compr Psychiatry, 2015 [38] | Single-center, cross-sectional | Turkey | | | 80 (+ 80 healthy controls) | AS |
| Espahbodi S, et al. Clin Exp Rheumatol, 2017 [40] | Single-center, cross-sectional | UK | | | 490 | axSpA |
| Gok K, et al. J Clin Rheumatol, 2018 [51] | Single-center, cross-sectional | Turkey | | | 185 | axSpA |
| Gossec L, et al. Joint Bone Spine, 2016 [52] | Multicenter, prospective, observational cohort | France | | | 486 | axSpA |
| Günaydin R, et al. Clin Rheumatol, 2009 [54] | Single-center, cross-sectional | Turkey | | | 62 | AS |
| Haywood KL, et al. Rheumatology (Oxford), 2014 [57] | Multicenter, cross-sectional | UK | | | 612 | AS |
| Heiberg T, et al. Ann Rheum Dis, 2011 [60] | Post hoc, exploratory analysis | Norway | | | 237 | AS |
| Ibn Yacoub Y, et al. Clin Rheumatol, 2010 [63] | Single-center, cross-sectional | Morocco | | | 100 | AS |
| Law L, et al. Arthritis Res Ther, 2018 [75] | Multicenter, case-control | Sweden | | | 210 (+ 1055 healthy volunteers) | AS |
| Morton L, et al. Arthritis Care Res (Hoboken), 2022 [86] | Multicenter, cross-sectional | Scotland | | | 718 | axSpA |
| Mustur D, et al. Srp Arh Celok Lek, 2009 [87] | Single-center, cross-sectional | Montenegro | | | 74 | AS |
| Nikiphorou E, et al. Rheumatology (Oxford), 2022 [90] | Multicenter, prospective, observational cohort | France | | | 704 | Early axSpA |
| Rezvani A, et al. Mod Rheumatol, 2014 [104] | Cross-sectional | Turkey | | | 421 | AS |
| Rostom S, et al. Rheumatol Int, 2013 [107] | Cross-sectional | Morocco | | | 110 | AS |
| Schneeberger EE, et al. Clin Rheumatol, 2015 [114] | Case-control | Argentina | | | 64 (+ 95 controls) | AS |
| Stebbings SM, et al. Clin Rheumatol, 2014 [121] | Single-center, cross-sectional | New Zealand | | | 67 | axSpA |
| Strand V, et al. J Clin Rheumatol, 2021 [122] | Multinational, cross-sectional | North America, Europe, Asia Pacific, Middle East | | | 705 | AS |
| Tang H, et al. Rheumatol Adv Pract, 2023 [159] | Systematic review  and meta-analysis | Global (Asia, America, UK, Europe, Africa) | | | 7893 | axSpA |
| Turan Y, et al. Rheumatol Int, 2007 [128] | Single-center, cross-sectional | Turkey | | | 68 | AS |
| Tymms K, et al. Clin Rheumatol, 2022 [130] | Multicenter, cross-sectional | Australia | | | 495 | AS |
| Ulus Y, et al. Clin Rheumatol, 2019 [132] | Single-center, cross-sectional,  case-control | Turkey | | | 61 (+ 40 controls) | AS |
| van Genderen S, et al. J Clin Rheumatol, 2014 [138] | Case-control study | The Netherlands | | | 24 (+ 24 controls) | AS |
| Wadeley A, et al. Clin Rheumatol, 2018 [144] | Single-center, cross-sectional | UK | | | 659 | axSpA |
| Yılmaz O, et al. Rheumatol Int, 2013 [148] | Single-center, prospective, observational | Turkey | | | 74 | AS |
| Žagar I, et al. Psychiatr Danub, 2021 [150] | Single-center, cross-sectional | Croatia | | | 150 | AS |
| Zhou W, et al. Clin Rheumatol, 2020 [152] | Single-center, cross-sectional | China | | | 150 | AS |

AS: ankylosing spondylitis; axSpA: axial spondyloarthritis.

| Table S9. Articles identified in the literature investigating physical function in association with psychosocial outcomes (*N* = 118) | | | | | | |
| --- | --- | --- | --- | --- | --- | --- |
| Publication, year [Ref] | **Study type** | **Location** | | **Number of patients** | | **Disease** |
| Aggarwal R, Malaviya AN. Clin Rheumatol, 2009 [1] | Single-center, prospective cohort | | India | | 70 | AS |
| Aissaoui N, et al. Rheumatol Int, 2012 [2] | Cross-sectional | | Morocco | | 110 | AS |
| Alkan BM, et al. Mod Rheumatol, 2013 [3] | Cross-sectional | | Turkey | | 110 (+ 40 healthy volunteers) | AS |
| Ariza-Ariza R, et al. J Rheumatol, 2009 [4] | Cross-sectional | | Spain | | 699 | AS |
| Atar E, Askin A. Int J Rheum Dis, 2020 [5] | Cross-sectional | | Turkey | | 80 | AS |
| Aykurt Karlıbel İ, et al. Aging Male, 2019 [6] | Prospective, observational cohort | | Turkey | | 67 | AS |
| Bakland G, et al. J Rheumatol, 2011 [8] | Single-center, prospective, observational cohort | | Norway | | 360 | AS |
| Bal S, et al. Rheumatol Int, 2011 [9] | Single-center, prospective, observational | | Turkey | | 37 (+ 67 healthy controls) | AS |
| Batmaz İ, et al. Rheumatol Int, 2013 [11] | Single-center, cross-sectional | | Turkey | | 80 (+ 52 controls) | AS |
| Baysal O, et al. Rheumatol Int, 2011 [12] | Multicenter, cross-sectional | | Turkey | | 243 (+ 118 healthy controls) | AS |
| Bedaiwi M, et al. J Rheumatol, 2015 [13] | Longitudinal cohort | | Canada | | 681 | axSpA |
| Bodur H, et al. Qual Life Res, 2011 [14] | Single-center, cross-sectional | | Turkey | | 962 | AS |
| Boonen A, et al. J Rheumatol, 2015 [15] | Prospective, multicenter, open-label, observational cohort | | Belgium | | 80 | AS |
| Boonen A, et al. Rheumatology (Oxford), 2018 [16] | Prospective, multicenter, open-label, observational cohort | | Belgium | | 75 | AS |
| Cakar E, et al. Clin Rheumatol, 2007 [18] | Double-center, cross-sectional | | Turkey | | 53 | AS |
| Cakar E, et al. Clin Rheumatol, 2009 [19] | Prospective, observational | | Turkey | | 121 | AS |
| Carvalho PD, et al. Arthritis Care Res (Hoboken), 2022 [20] | Prospective, multicenter, observational cohort | | France | | 644 | Early axSpA |
| Cengiz G, et al. Int J Rheum Dis, 2023 [21] | Single-center, cross-sectional | | Turkey | | 110 | axSpA |
| Chen CH, et al. Int J Rheum Dis, 2020 [22] | Single-center, cross-sectional | | Taiwan | | 105 | AS |
| Chen CH, et al. Clin Rheumatol, 2013 [23] | Single-center, cross-sectional | | Taiwan | | 75 | AS |
| Chung DXY, et al. BMJ Open, 2023 [26] | Single-center, cross-sectional | | Singapore | | 296 | axSpA |
| Chung HY, et al. Ann Rheum Dis, 2012 [27] | Multicenter, cross-sectional | | France | | 647 | axSpA |
| Connolly D, et al. Occup Ther Int, 2019 [28] | Single-center, cross-sectional | | Ireland | | 50 | AS |
| Cunha RN, et al. ARP Rheumatol, 2022 [29] | Multicenter, cross-sectional | | Portugal | | 1995 | axSpA |
| Demir SE, et al. Rheumatol Int, 2013 [32] | Single-center, cross-sectional | | Turkey | | 23 (+ 27 healthy controls) | AS |
| Dhakad U, et al. Int J Rheum Dis, 2015 [33] | Single-center, prospective, case-control study | | India | | 100 (+ 100 healthy controls) | AS |
| Dincer U, et al. Tohoku J Exp Med, 2007 [34] | Single-center, cross-sectional | | Turkey | | 36 (+ 34 controls) | AS |
| Dincer U, et al. Rheumatol Int, 2007 [35] | Single-center, cross-sectional | | Turkey | | 65 (+ 45 healthy controls) | AS |
| Dülger S, et al. J Clin Rheumatol, 2019 [36] | Single-blind, controlled, observational | | Turkey | | 92 | AS |
| Durcan L, et al. J Rheumatol, 2012 [37] | Single-center, cross-sectional | | Ireland | | 46 | AS |
| Durmus D, et al. Compr Psychiatry, 2015 [38] | Single-center, cross-sectional | | Turkey | | 80 (+ 80 healthy controls) | AS |
| Elolemy G, et al. Curr Rheumatol Rev, 2020 [39] | Multicenter, cross-sectional | | Kuwait and Saudi Arabia | | 47 | AS |
| Espahbodi S, et al. Clin Exp Rheumatol, 2017 [40] | Single-center, cross-sectional | | UK | | 490 | axSpA |
| Fernández-Carballido C, et al. Arthritis Care Res (Hoboken), 2020 [42] | Multicenter, cross-sectional | | Spain | | 738  (+ 721 with PsA) | AS |
| Fernández-Carballido C, et al; Esperanza Study Group. Arthritis Care Res (Hoboken), 2017 [43] | Multicenter, cross-sectional | | Spain | | 259 | Early axSpA |
| Fongen C, et al. Musculoskeletal Care, 2015 [44] | Single-center, cross-sectional | | Norway | | 148 (+ 133 controls) | AS |
| Frede N, et al. RMD Open, 2023 [46] | Single-center, retrospective, cross-sectional | | Austria | | 162  (+ 168 with PsA) | axSpA |
| Garrido-Cumbrera M, et al. Arthritis Care Res (Hoboken), 2021 [47] | Multicenter, cross-sectional | | 13 European countries | | 2695 | axSpA |
| Garrido-Cumbrera M, et al. J Rheumatol, 2022 [48] | Cross-sectional | | Spain | | 653 | axSpA |
| Garrido-Cumbrera M. J Rheumatol, 2019 [49] | Cross-sectional | | Spain | | 474 | axSpA |
| Garrido-Cumbrera M, et al; EMAS working group. RMD Open, 2021 [50] | Multicenter, cross-sectional | | 12 European countries | | 2166 | axSpA |
| Gözüküçük M, et al. Clin Exp Rheumatol, 2022 [53] | Single-center, cross-sectional, case-control | | Turkey | | 98 (+ 99 healthy controls) | axSpA |
| Günaydin R, et al. Clin Rheumatol, 2009 [54] | Single-center, cross-sectional | | Turkey | | 62 | AS |
| Hakkou J, et al. J Clin Rheumatol, 2011 [55] | Single-center, cross-sectional | | Morocco | | 110 | AS |
| Hakkou J, et al. Rheumatol Int, 2013 [56] | Single-center, cross-sectional | | Morocco | | 110 | AS |
| Haywood KL, et al. Rheumatology (Oxford), 2014 [57] | Multicenter, cross-sectional | | UK | | 612 | AS |
| Healey EL, et al. Scand J Rheumatol, 2011 [58] | Multicenter, cross-sectional | | UK | | 612 | AS |
| Healey EL, et al. Rheumatology (Oxford), 2009 [59] | Multicenter, cross-sectional | | UK | | 612 | AS |
| Heiberg T, et al. Ann Rheum Dis, 2011 [60] | Post hoc, exploratory analysis | | Norway | | 237 | AS |
| Huang JC, et al. BMC Musculoskelet Disord, 2017 [61] | Retrospective case series | | China | | 245 | AS |
| Hwang MC, et al. Rheumatol Int, 2020 [62] | Multicenter, prospective, observational cohort | | USA | | 991 | AS |
| Ibn Yacoub Y, et al. Clin Rheumatol, 2010 [63] | Single-center, cross-sectional | | Morocco | | 100 | AS |
| Ibn Yacoub Y, et al. Clin Rheumatol, 2011 [64] | Single-center, cross-sectional | | Morocco | | 100 | AS |
| Jiang Y, et al. Clin Rheumatol, 2018 [65] | Multicenter, cross-sectional | | China | | 2772 (+ unknown no. of volunteers) | AS |
| Kieskamp SC, et al. Semin Arthritis Rheum, 2022 [70] | Prospective, observational cohort | | The Netherlands | | 182 | axSpA |
| Kiltz U, et al. RMD Open, 2023 [71] | Multicenter, cross-sectional | | Germany | | 770 | axSpA |
| Landewé R, et al. Ann Rheum Dis, 2009 [74] | Multicenter, prospective, observational cohort | | The Netherlands, Belgium, France | | 217 | AS |
| Law L, et al. Arthritis Res Ther, 2018 [75] | Multicenter, case-control | | Sweden | | 210 (+ 1055 healthy volunteers) | AS |
| Li Y, et al. Arthritis Res Ther, 2012 [76] | Multicenter, case-control | | China | | 314 (+ 102 healthy controls) | AS |
| Lu MC, et al. BMJ Open, 2019 [77] | Single-center, cross-sectional | | Taiwan | | 265 | AS |
| Machado P, et al. Ann Rheum Dis, 2011 [78] | Post hoc, exploratory, cross-sectional analysis | | Europe and USA | | 224 | AS |
| Magrey M, et al. ACR Open Rheumatol, 2023 [79] | Multicenter, cross-sectional | | USA | | 228 | axSpA |
| Maguire S, et al. Scand J Rheumatol, 2022 [80] | Multicenter, cross-sectional | | Ireland | | 759 | axSpA |
| Maguire S, et al. J Rheumatol, 2022 [81] | Multicenter,  cross-sectional | | Ireland | | 753 | axSpA |
| Maksymowych WP, et al. J Rheumatol, 2010 [82] | Multicenter, randomized, double-blind,  placebo-controlled | | USA | | 315 | AS |
| Mattey DL, et al. J Rheumatol, 2011 [83] | Multicenter, cross-sectional | | UK | | 606 | AS |
| Mease PJ, et al. J Rheumatol, 2021 [84] | Multicenter, prospective, observational cohort | | USA | | 498 | axSpA |
| Mease PJ, et al. ACR Open Rheumatol, 2020 [155] | Multicenter, prospective, observational | | USA | | 477 | axSpA |
| Mlcoch T, et al. Expert Rev Pharmacoecon Outcomes Res, 2017 [85] | Multicenter, prospective, observational cohort | | Czech Republic | | 313 | AS |
| Morton L, et al. Arthritis Care Res (Hoboken), 2022 [86] | Multicenter, cross-sectional | | Scotland | | 718 | axSpA |
| Mustur D, et al. Srp Arh Celok Lek, 2009 [87] | Single-center, cross-sectional | | Montenegro | | 74 | AS |
| Nam B, et al. Qual Life Res, 2021 [88] | Single-center, cross-sectional | | Republic of Korea | | 211 | AS |
| Nie A, et al. Clin Rheumatol, 2018 [89] | Single-center, cross-sectional | | China | | 281 | AS |
| Nikiphorou E, et al. Arthritis Care Res (Hoboken), 2018 [91] | Multicenter, cross-sectional | | Africa, Americas, Asia, Europe | | 3370 | axSpA |
| O'Dwyer T, et al. Rheumatol Int, 2015 [92] | Single-center, cross-sectional, controlled | | Ireland | | 39 (+ 39 controls) | AS |
| O'Dwyer T, et al. Physiotherapy, 2016 [93] | Single-center, cross-sectional, controlled | | Ireland | | 39 (+ 39 controls) | AS |
| Öksüz E, et al. Perspect Psychiatr Care, 2021 [95] | Single-center, cross-sectional | | Turkey | | 119 | AS |
| Ortancil O, et al. J Clin Rheumatol, 2010 [97] | Single-center, cross-sectional | | Turkey | | 29 (+ 20 controls) | AS |
| Ozdemir O. Rheumatol Int, 2011 [98] | Single-center, cross-sectional | | Turkey | | 48 | AS |
| Poddubnyy D, et al. Ann Rheum Dis, 2013 [101] | Multicenter, prospective cohort | | Germany | | 210 | axSpA |
| Rafia R, et al. Clin Exp Rheumatol, 2012 [102] | Multicenter, cross-sectional | | UK | | 612 | AS |
| Redeker I, et al. Ann Rheum Dis, 2018 [103] | Multicenter, cross-sectional | | Germany | | 1736 | axSpA |
| Rezvani A, et al. Mod Rheumatol, 2014 [104] | Cross-sectional | | Turkey | | 421 | AS |
| Rohde G, et al. Qual Life Res, 2020 [105] | Multicenter, prospective, observational cohort | | Norway | | 380 | axSpA |
| Rosas J, et al; and the AIRE-MB Group. Clin Exp Rheumatol, 2017 [106] | Single-center, cross-sectional | | Spain | | 57 | AS |
| Rostom S, et al. Rheumatol Int, 2013 [107] | Cross-sectional | | Morocco | | 110 | AS |
| Sağ S, et al. J Back Musculoskelet Rehabil, 2018 [108] | Single-center, cross-sectional | | Turkey | | 50 (+ 30 controls) | AS |
| Sakellariou GT, et al. Rheumatol Int, 2015 [109] | Single-center, cross-sectional | | Greece | | 106 | AS |
| Sariyildiz MA, et al. Rheumatol Int, 2013 [111] | Multicenter, cross-sectional | | Turkey | | 70 (+ 60 controls) | AS |
| Sariyildiz MA, et al. Int J Impot Res, 2013 [112] | Multicenter, cross-sectional | | Turkey | | 37 (+ 33 controls) | AS |
| Schmalz G, et al. Clin Oral Investig, 2018 [113] | Single-center, cross-sectional | | Germany | | 50 (+ 50 controls) | AS |
| Schneeberger EE, et al. Clin Rheumatol, 2015 [114] | Case-control | | Argentina | | 64 (+ 95 controls) | AS |
| Shahlaee A, et al. Clin Rheumatol, 2015 [115] | Single-center, cross-sectional | | Iran | | 320 | AS |
| Shen B, et al. Rheumatol Int, 2013 [116] | Single-center, cross-sectional | | China | | 103 (+ 121 controls) | AS |
| Shen B, et al. Psychol Psychother, 2014 [117] | Single-center, cross-sectional | | China | | 103 (+ 121 controls) | AS |
| Stebbings SM, et al. Clin Rheumatol, 2014 [121] | Single-center, cross-sectional | | New Zealand | | 67 | axSpA |
| Swinnen TW, et al. Arthritis Res Ther, 2018 [123] | Single-center, cross-sectional | | Belgium | | 170 | axSpA |
| Tam LS, et al. J Rheumatol, 2007 [124] | Multicenter, cross-sectional | | Hong Kong | | 314 | AS |
| Tarhan F, et al. Int J Androl, 2012 [125] | Single-center, cross-sectional | | Turkey | | 50 (+ 50 healthy controls) | AS |
| Taser B, et al. Acta Reumatol Port, 2017 [126] | Single-center, cross-sectional | | Turkey | | 100 | AS |
| Tu L, et al. Clin Exp Rheumatol, 2014 [127] | Single-center, cross-sectional | | China | | 257 | AS |
| Turan Y, et al. Rheumatol Int, 2007 [128] | Single-center, cross-sectional | | Turkey | | 68 | AS |
| Turan Y, et al. Rheumatol Int, 2007 [129] | Cross-sectional | | Turkey | | 46 | AS |
| Uludag M, et al. Rheumatol Int, 2012 [131] | Single-center, cross-sectional | | Turkey | | 25 (+ their spouses + 25 healthy controls) | AS |
| Ulus Y, et al. Clin Rheumatol, 2019 [132] | Single-center, cross-sectional,  case-control | | Turkey | | 61 (+ 40 controls) | AS |
| Urkmez B, Keskin Y. Mod Rheumatol, 2020 [133] | Cross-sectional | | Turkey | | 150 (+ 80 controls) | AS |
| van der Heijde D, et al. Rheumatology (Oxford), 2016 [134] | Post hoc analysis of a multicenter, Phase 3 RCT | | Australia, Belgium,  Canada, Czech Republic, France, Germany, Spain, the Netherlands,  UK, USA | | 179 | TNFi-IR  nr-axSpA |
| van der Horst-Bruinsma IE, et al. Ann Rheum Dis, 2013 [135] | Post hoc, exploratory analysis | | The Netherlands | | 1283 | AS |
| van Genderen S, et al. J Clin Rheumatol, 2014 [138] | Case-control study | | The Netherlands | | 24 (+ 24 controls) | AS |
| van Lunteren M, et al. J Rheumatol, 2018 [139] | Multinational, prospective cohort | | Europe | | 161 | Early axSpA |
| Vesović-Potić V, et al. Rheumatol Int, 2009 [142] | Single-center, cross-sectional | | Montenegro | | 74 | AS |
| Wadeley A, et al. Clin Rheumatol, 2018 [144] | Single-center, cross-sectional | | UK | | 659 | axSpA |
| Ward MM, et al. Arthritis Rheum, 2008 [146] | Multicenter, cross-sectional | | USA | | 591 | AS |
| Yılmaz O, et al. Rheumatol Int, 2013 [148] | Single-center, prospective, observational | | Turkey | | 74 | AS |
| Yüce E, et al. Agri, 2023 [149] | Single-center, cross-sectional,  case-control | | Turkey | | 100 (+ 100 controls) | AS |
| Žagar I, et al. Psychiatr Danub, 2021 [150] | Single-center, cross-sectional | | Croatia | | 150 | AS |
| Zhang H, et al. Clin Rheumatol, 2018 [151] | Multicenter, cross-sectional | | China | | 1178 | AS |
| Zhou W, et al. Clin Rheumatol, 2020 [152] | Single-center, cross-sectional | | China | | 150 | AS |

AS: ankylosing spondylitis; axSpA: axial spondyloarthritis; nr-axSpA: non-radiographic axSpA; PsA: psoriatic arthritis; TNFi-IR: inadequate response to tumor necrosis factor inhibitor.

| Table S10. Articles identified in the literature investigating overall functioning and health in association with psychosocial outcomes (*N* = 129) | | | | | | | |
| --- | --- | --- | --- | --- | --- | --- | --- |
| Publication, year [Ref] | **Study type** | | **Location** | **Number of patients** | | **Disease** | |
| Aissaoui N, et al. Rheumatol Int, 2012 [2] | Cross-sectional | Morocco | | | 110 | | AS |
| Alkan BM, et al. Mod Rheumatol, 2013 [3] | Cross-sectional | Turkey | | | 110 (+ 40 healthy volunteers) | | AS |
| Ariza-Ariza R, et al. J Rheumatol, 2009 [4] | Cross-sectional | Spain | | | 699 | | AS |
| Atar E, Askin A. Int J Rheum Dis, 2020 [5] | Cross-sectional | Turkey | | | 80 | | AS |
| Aykurt Karlıbel İ, et al. Aging Male, 2019 [6] | Prospective, observational cohort | Turkey | | | 67 | | AS |
| Aykurt Karlıbel I, Kasapoğlu Aksoy M. Ir J Med Sci, 2023 [7] | Cross-sectional | Turkey | | | 82 (+ 40 healthy volunteers) | | axSpA |
| Bal S, et al. Rheumatol Int, 2011 [9] | Single-center, prospective, observational | Turkey | | | 37 (+ 67 healthy controls) | | AS |
| Batmaz İ, et al. Rheumatol Int, 2013 [11] | Single-center, cross-sectional | Turkey | | | 80 (+ 52 controls) | | AS |
| Baysal O, et al. Rheumatol Int, 2011 [12] | Multicenter, cross-sectional | Turkey | | | 243 (+ 118 healthy controls) | | AS |
| Bedaiwi M, et al. J Rheumatol, 2015 [13] | Longitudinal cohort | Canada | | | 681 | | axSpA |
| Bodur H, et al. Qual Life Res, 2011 [14] | Single-center, cross-sectional | Turkey | | | 962 | | AS |
| Brophy S, et al. Semin Arthritis Rheum, 2013 [17] | Prospective, multicenter, observational | UK | | | 385 | | AS |
| Cakar E, et al. Clin Rheumatol, 2007 [18] | Double-center, cross-sectional | Turkey | | | 53 | | AS |
| Cakar E, et al. Clin Rheumatol, 2009 [19] | Prospective, observational | Turkey | | | 121 | | AS |
| Carvalho PD, et al. Arthritis Care Res (Hoboken), 2022 [20] | Prospective, multicenter, observational cohort | France | | | 644 | | Early axSpA |
| Cengiz G, et al. Int J Rheum Dis, 2023 [21] | Single-center, cross-sectional | Turkey | | | 110 | | axSpA |
| Chen CH, et al. Int J Rheum Dis, 2020 [22] | Single-center, cross-sectional | Taiwan | | | 105 | | AS |
| Chen HH, et al. PLoS One, 2020 [24] | Single-center, cross-sectional | Taiwan | | | 307 | | AS |
| Chen MH, et al. Clin Rheumatol, 2018 [25] | Multicenter, retrospective | Taiwan | | | 120 | | AS |
| Chung DXY, et al. BMJ Open, 2023 [26] | Single-center, cross-sectional | Singapore | | | 296 | | axSpA |
| Chung HY, et al. Ann Rheum Dis, 2012 [27] | Multicenter, cross-sectional | France | | | 647 | | axSpA |
| Connolly D, et al. Occup Ther Int, 2019 [28] | Single-center, cross-sectional | Ireland | | | 50 | | AS |
| D'Angelo S, et al. Clin Exp Rheumatol, 2019 [30] | Multicenter, prospective, observational | Italy | | | 512 | | axSpA |
| D'Angelo S, et al. Clin Exp Rheumatol, 2021 [31] | Multicenter, prospective, observational | Italy | | | 512 | | axSpA |
| Demir SE, et al. Rheumatol Int, 2013 [32] | Single-center, cross-sectional | Turkey | | | 23 (+ 27 healthy controls) | | AS |
| Dhakad U, et al. Int J Rheum Dis, 2015 [33] | Single-center, prospective, case-control study | India | | | 100 (+ 100 healthy controls) | | AS |
| Dincer U, et al. Tohoku J Exp Med, 2007 [34] | Single-center, cross-sectional | Turkey | | | 36 (+ 34 controls) | | AS |
| Dincer U, et al. Rheumatol Int, 2007 [35] | Single-center, cross-sectional | Turkey | | | 65 (+ 45 healthy controls) | | AS |
| Dülger S, et al. J Clin Rheumatol, 2019 [36] | Single-blind, controlled, observational | Turkey | | | 92 | | AS |
| Durcan L, et al. J Rheumatol, 2012 [37] | Single-center, cross-sectional | Ireland | | | 46 | | AS |
| Durmus D, et al. Compr Psychiatry, 2015 [38] | Single-center, cross-sectional | Turkey | | | 80 (+ 80 healthy controls) | | AS |
| Elolemy G, et al. Curr Rheumatol Rev, 2020 [39] | Multicenter, cross-sectional | Kuwait and Saudi Arabia | | | 47 | | AS |
| Espahbodi S, et al. Clin Exp Rheumatol, 2017 [40] | Single-center, cross-sectional | UK | | | 490 | | axSpA |
| Fernández-Carballido C, et al; Esperanza Study Group. Arthritis Care Res (Hoboken), 2017 [43] | Multicenter, cross-sectional | Spain | | | 259 | | Early axSpA |
| Forejtová S, et al. Clin Rheumatol, 2008 [45] | Single-center, cross-sectional | Czech Republic | | | 1001 | | AS |
| Frede N, et al. RMD Open, 2023 [46] | Single-center, retrospective, cross-sectional | Austria | | | 162  (+ 168 with PsA) | | axSpA |
| Garrido-Cumbrera M, et al. Arthritis Care Res (Hoboken), 2021 [47] | Multicenter, cross-sectional | 13 European countries | | | 2695 | | axSpA |
| Garrido-Cumbrera M, et al. J Rheumatol, 2022 [48] | Cross-sectional | Spain | | | 653 | | axSpA |
| Garrido-Cumbrera M. J Rheumatol, 2019 [49] | Cross-sectional | Spain | | | 474 | | axSpA |
| Garrido-Cumbrera M, et al; EMAS working group. RMD Open, 2021 [50] | Multicenter, cross-sectional | 12 European countries | | | 2166 | | axSpA |
| Gok K, et al. J Clin Rheumatol, 2018 [51] | Single-center, cross-sectional | Turkey | | | 185 | | axSpA |
| Gossec L, et al. Joint Bone Spine, 2016 [52] | Multicenter, prospective, observational cohort | France | | | 486 | | axSpA |
| Gözüküçük M, et al. Clin Exp Rheumatol, 2022 [53] | Single-center, cross-sectional, case-control | Turkey | | | 98 (+ 99 healthy controls) | | axSpA |
| Günaydin R, et al. Clin Rheumatol, 2009 [54] | Single-center, cross-sectional | Turkey | | | 62 | | AS |
| Hakkou J, et al. J Clin Rheumatol, 2011 [55] | Single-center, cross-sectional | Morocco | | | 110 | | AS |
| Hakkou J, et al. Rheumatol Int, 2013 [56] | Single-center, cross-sectional | Morocco | | | 110 | | AS |
| Haywood KL, et al. Rheumatology (Oxford), 2014 [57] | Multicenter, cross-sectional | UK | | | 612 | | AS |
| Healey EL, et al. Scand J Rheumatol, 2011 [58] | Multicenter, cross-sectional | UK | | | 612 | | AS |
| Healey EL, et al. Rheumatology (Oxford), 2009 [59] | Multicenter, cross-sectional | UK | | | 612 | | AS |
| Heiberg T, et al. Ann Rheum Dis, 2011 [60] | Post hoc, exploratory analysis | Norway | | | 237 | | AS |
| Huang JC, et al. BMC Musculoskelet Disord, 2017 [61] | Retrospective case series | China | | | 245 | | AS |
| Hwang MC, et al. Rheumatol Int, 2020 [62] | Multicenter, prospective, observational cohort | USA | | | 991 | | AS |
| Ibn Yacoub Y, et al. Clin Rheumatol, 2010 [63] | Single-center, cross-sectional | Morocco | | | 100 | | AS |
| Ibn Yacoub Y, et al. Clin Rheumatol, 2011 [64] | Single-center, cross-sectional | Morocco | | | 100 | | AS |
| Jiang Y, et al. Clin Rheumatol, 2018 [65] | Multicenter, cross-sectional | China | | | 2772 (+ unknown no. of volunteers) | | AS |
| Jiang Y, et al. Clin Rheumatol, 2015 [66] | Multicenter, cross-sectional | China | | | 683 | | AS |
| Kankaya H, et al. J Transcult Nurs, 2023 [67] | Single-center, cross-sectional | Turkey | | | 101 | | AS |
| Karatekin BD, et al. J Coll Physicians Surg Pak, 2023 [68] | Single-center, prospective, observational cohort | Turkey | | | 41 | | AS |
| Kedra J, et al. J Rheumatol, 2022 [69] | Multicenter, cross-sectional | France | | | 638 | | axSpA |
| Kieskamp SC, et al. Semin Arthritis Rheum, 2022 [70] | Prospective, observational cohort | The Netherlands | | | 182 | | axSpA |
| Kiltz U, et al. RMD Open, 2023 [71] | Multicenter, cross-sectional | Germany | | | 770 | | axSpA |
| Korotaeva T, et al. Clin Rheumatol, 2021 [72] | Cross-sectional | USA | | | 487 | | AS |
|  |  | CEE | | | 922 | |  |
| Kydd AS, et al. Rheumatology (Oxford), 2015 [73] | Retrospective, longitudinal cohort | Australia | | | 422 | | AS |
| Law L, et al. Arthritis Res Ther, 2018 [75] | Multicenter, case-control | Sweden | | | 210 (+ 1055 healthy volunteers) | | AS |
| Li Y, et al. Arthritis Res Ther, 2012 [76] | Multicenter, case-control | China | | | 314 (+ 102 healthy controls) | | AS |
| Lu MC, et al. BMJ Open, 2019 [77] | Single-center, cross-sectional | Taiwan | | | 265 | | AS |
| Machado P, et al. Ann Rheum Dis, 2011 [78] | Post hoc, exploratory, cross-sectional analysis | Europe and USA | | | 224 | | AS |
| Magrey M, et al. ACR Open Rheumatol, 2023 [79] | Multicenter, cross-sectional | USA | | | 228 | | axSpA |
| Maguire S, et al. Scand J Rheumatol, 2022 [80] | Multicenter, cross-sectional | Ireland | | | 759 | | axSpA |
| Maguire S, et al. J Rheumatol, 2022 [81] | Multicenter,  cross-sectional | Ireland | | | 753 | | axSpA |
| Maksymowych WP, et al. J Rheumatol, 2010 [82] | Multicenter, randomized, double-blind,  placebo-controlled | USA | | | 315 | | AS |
| Mattey DL, et al. J Rheumatol, 2011 [83] | Multicenter, cross-sectional | UK | | | 606 | | AS |
| Mease PJ, et al. J Rheumatol, 2021 [84] | Multicenter, prospective, observational cohort | USA | | | 498 | | axSpA |
| Mlcoch T, et al. Expert Rev Pharmacoecon Outcomes Res, 2017 [85] | Multicenter, prospective, observational, cohort | Czech Republic | | | 313 | | AS |
| Morton L, et al. Arthritis Care Res (Hoboken), 2022 [86] | Multicenter, cross-sectional | Scotland | | | 718 | | axSpA |
| Mustur D, et al. Srp Arh Celok Lek, 2009 [87] | Single-center, cross-sectional | Montenegro | | | 74 | | AS |
| Nam B, et al. Qual Life Res, 2021 [88] | Single-center, cross-sectional | Republic of Korea | | | 211 | | AS |
| Nie A, et al. Clin Rheumatol, 2018 [89] | Single-center, cross-sectional | China | | | 281 | | AS |
| Nikiphorou E, et al. Rheumatology (Oxford), 2022 [90] | Multicenter, prospective, observational cohort | France | | | 704 | | Early axSpA |
| Nikiphorou E, et al. Arthritis Care Res (Hoboken), 2018 [91] | Multicenter, cross-sectional | Africa, Americas, Asia, Europe | | | 3370 | | axSpA |
| O'Dwyer T, et al. Rheumatol Int, 2015 [92] | Single-center, cross-sectional, controlled | Ireland | | | 39 (+ 39 controls) | | AS |
| O'Dwyer T, et al. Physiotherapy, 2016 [93] | Single-center, cross-sectional, controlled | Ireland | | | 39 (+ 39 controls) | | AS |
| Öğüt TS, et al. Clin Rheumatol, 2023 [94] | Single-center, cross-sectional | Turkey | | | 175 | | axSpA |
| Öksüz E, et al. Perspect Psychiatr Care, 2021 [95] | Single-center, cross-sectional | Turkey | | | 119 | | AS |
| Omar M, et al. J Affect Disord, 2023 [96] | Retrospective, population-based, cross-sectional | Israel | | | 5825 (+ 25 984 controls) | | AS |
| Ortancil O, et al. J Clin Rheumatol, 2010 [97] | Single-center, cross-sectional | Turkey | | | 29 (+ 20 controls) | | AS |
| Ozdemir O. Rheumatol Int, 2011 [98] | Single-center, cross-sectional | Turkey | | | 48 | | AS |
| Park JS, et al. Sci Rep, 2019 [99] | Multicenter, prospective, observational cohort | Republic of Korea | | | 11 465 (+  57 325 controls) | | AS |
| Redeker I, et al. Ann Rheum Dis, 2018 [103] | Multicenter, cross-sectional | Germany | | | 1736 | | axSpA |
| Rezvani A, et al. Mod Rheumatol, 2014 [104] | Cross-sectional | Turkey | | | 421 | | AS |
| Rohde G, et al. Qual Life Res, 2020 [105] | Multicenter, prospective, observational cohort | Norway | | | 380 | | axSpA |
| Rostom S, et al. Rheumatol Int, 2013 [107] | Cross-sectional | Morocco | | | 110 | | AS |
| Sağ S, et al. J Back Musculoskelet Rehabil, 2018 [108] | Single-center, cross-sectional | Turkey | | | 50 (+ 30 controls) | | AS |
| Santana T, et al. Int Braz J Urol, 2017 [110] | Single-center, cross-sectional | Brazil | | | 40 (+ 40 controls) | | AS |
| Sariyildiz MA, et al. Rheumatol Int, 2013 [111] | Multicenter, cross-sectional | Turkey | | | 70 (+ 60 controls) | | AS |
| Sariyildiz MA, et al. Int J Impot Res, 2013 [112] | Multicenter, cross-sectional | Turkey | | | 37 (+ 33 controls) | | AS |
| Schmalz G, et al. Clin Oral Investig, 2018 [113] | Single-center, cross-sectional | Germany | | | 50 (+ 50 controls) | | AS |
| Schneeberger EE, et al. Clin Rheumatol, 2015 [114] | Case-control | Argentina | | | 64 (+ 95 controls) | | AS |
| Shahlaee A, et al. Clin Rheumatol, 2015 [115] | Single-center, cross-sectional | Iran | | | 320 | | AS |
| Shen B, et al. Rheumatol Int, 2013 [116] | Single-center, cross-sectional | China | | | 103 (+ 121 controls) | | AS |
| Shen B, et al. Psychol Psychother, 2014 [117] | Single-center, cross-sectional | China | | | 103 (+ 121 controls) | | AS |
| Shen CC, et al. J Rheumatol, 2016 [118] | Multicenter, retrospective cohort | Taiwan | | | 2331 (+ 9324 controls) | | AS |
| Sieper J, et al. Clin Exp Rheumatol, 2016 [119] | Multicenter, cross-sectional | Europe (Germany, France, Spain, Italy, UK) | | | 631 | | nr-axSpA |
| Stebbings SM, et al. Clin Rheumatol, 2014 [121] | Single-center, cross-sectional | New Zealand | | | 67 | | axSpA |
| Strand V, et al. J Clin Rheumatol, 2021 [122] | Multinational, cross-sectional | North America, Europe, Asia Pacific, Middle East | | | 705 | | AS |
| Swinnen TW, et al. Arthritis Res Ther, 2018 [123] | Single-center, cross-sectional | Belgium | | | 170 | | axSpA |
| Tarhan F, et al. Int J Androl, 2012 [125] | Single-center, cross-sectional | Turkey | | | 50 (+ 50 healthy controls) | | AS |
| Taser B, et al. Acta Reumatol Port, 2017 [126] | Single-center, cross-sectional | Turkey | | | 100 | | AS |
| Tu L, et al. Clin Exp Rheumatol, 2014 [127] | Single-center, cross-sectional | China | | | 257 | | AS |
| Turan Y, et al. Rheumatol Int, 2007 [128] | Single-center, cross-sectional | Turkey | | | 68 | | AS |
| Turan Y, et al. Rheumatol Int, 2007 [129] | Cross-sectional | Turkey | | | 46 | | AS |
| Uludag M, et al. Rheumatol Int, 2012 [131] | Single-center, cross-sectional | Turkey | | | 25 (+ their spouses + 25 healthy controls) | | AS |
| Ulus Y, et al. Clin Rheumatol, 2019 [132] | Single-center, cross-sectional case-control | Turkey | | | 61 (+ 40 controls) | | AS |
| Urkmez B, Keskin Y. Mod Rheumatol, 2020 [133] | Cross-sectional | Turkey | | | 150 (+ 80 controls) | | AS |
| van der Heijde D, et al. Rheumatology (Oxford), 2016 [134] | Post hoc analysis of a multicenter, Phase 3 RCT | Australia, Belgium,  Canada, Czech Republic, France, Germany, Spain, the Netherlands,  UK, USA | | | 179 | | TNFi-IR  nr-axSpA |
| van Genderen S, et al. Arthritis Care Res (Hoboken), 2016 [136] | Multicenter, cross-sectional | The Netherlands | | | 246 (+ 784 controls) | | AS |
| van Genderen S, et al. Arthritis Care Res (Hoboken), 2018 [137] | Multicenter  cross-sectional | The Netherlands | | | 246 (+ 510 controls) | | AS |
| van Genderen S, et al. J Clin Rheumatol, 2014 [138] | Case-control study | The Netherlands | | | 24 (+ 24 controls) | | AS |
| van Lunteren M, et al. J Rheumatol, 2018 [139] | Multinational, prospective cohort | Europe | | | 161 | | Early axSpA |
| Vergne-Salle P, et al. Nutrients, 2022 [141] | Single-center, non-interventional cohort | France | | | 235 | | axSpA |
| Vesović-Potić V, et al. Rheumatol Int, 2009 [142] | Single-center, cross-sectional | Montenegro | | | 74 | | AS |
| Vitturi BK, et al. Can J Neurol Sci, 2020 [143] | Single-center, cross-sectional,  case-control | Brazil | | | 40 (+ 40 controls) | | AS |
| Wadeley A, et al. Clin Rheumatol, 2018 [144] | Single-center, cross-sectional | UK | | | 659 | | axSpA |
| Walsh JA, et al. Clin Rheumatol, 2018 [145] | Retrospective, observational cohort | USA | | | 6679 (+ 19951 controls) | | AS |
| Wu JJ, et al. J Eur Acad Dermatol Venereol, 2017 [147] | Population-based cohort | USA | | | 1878 | | AS |
| Yılmaz O, et al. Rheumatol Int, 2013 [148] | Single-center, prospective, observational | Turkey | | | 74 | | AS |
| Yüce E, et al. Agri, 2023 [149] | Single-center, cross-sectional,  case-control | Turkey | | | 100 (+ 100 controls) | | AS |
| Žagar I, et al. Psychiatr Danub, 2021 [150] | Single-center, cross-sectional | Croatia | | | 150 | | AS |
| Zhou W, et al. Clin Rheumatol, 2020 [152] | Single-center, cross-sectional | China | | | 150 | | AS |

AS: ankylosing spondylitis; axSpA: axial spondyloarthritis; nr-axSpA: non-radiographic axSpA; PsA: psoriatic arthritis; TNFi-IR: inadequate response to tumor necrosis factor inhibitor.

**References**

1. Aggarwal R, Malaviya AN. Diagnosis delay in patients with ankylosing spondylitis: factors and outcomes–an Indian perspective. Clin Rheumatol 2009;28:327–31.

2. Aissaoui N, Rostom S, Hakkou J, Berrada Ghziouel K, Bahiri R, Abouqal R, *et al.* Fatigue in patients with ankylosing spondylitis: prevalence and relationships with disease-specific variables, psychological status, and sleep disturbance. Rheumatol Int 2012;32:2117–24.

3. Alkan BM, Fidan F, Erten Ş, Aksekili H, Alemdar A, Eroğlu E, *et al.* Fatigue and correlation with disease-specific variables, spinal mobility measures, and health-related quality of life in ankylosing spondylitis. Mod Rheumatol 2013;23:1101–7.

4. Ariza-Ariza R, Hernández-Cruz B, Collantes E, Batlle E, Fernández-Sueiro JL, Gratacós J, *et al.* Work disability in patients with ankylosing spondylitis. J Rheumatol 2009;36:2512–6.

5. Atar E, Askin A. Somatosensory dysfunction related neuropathic pain component affects disease activity, functional status and quality of life in ankylosing spondylitis. Int J Rheum Dis 2020;23:1656–63.

6. Aykurt Karlıbel İ, Dülger S, Kasapoğlu Aksoy M, Güzelsoy M, Türkoğlu AR, Altan L, *et al.* Effect of cigarette smoking on sexual functions, psychological factors, and disease activity in male patients with ankylosing spondylitis. Aging Male 2019;22:109–15.

7. Aykurt Karlıbel I, Kasapoğlu Aksoy M. The relationship between central sensitization and disease activity, quality of life, and sleep quality among patients with axial spondyloarthritis. Ir J Med Sci 2023;192:481–9.

8. Bakland G, Gran JT, Becker-Merok A, Nordvåg BY, Nossent JC. Work disability in patients with ankylosing spondylitis in Norway. J Rheumatol 2011;38:479–84.

9. Bal S, Bal K, Turan Y, Deniz G, Gürgan A, Berkit IK, *et al.* Sexual functions in ankylosing spondylitis. Rheumatol Int 2011;31:889–94.

10. Bandinelli F, Salvadorini G, Delle Sedie A, Riente L, Bombardieri S, Matucci-Cerinic M. Impact of gender, work, and clinical presentation on diagnostic delay in Italian patients with primary ankylosing spondylitis. Clin Rheumatol 2016;35:473–8.

11. Batmaz İ, Sarıyıldız MA, Dilek B, Bez Y, Karakoç M, Çevik R. Sleep quality and associated factors in ankylosing spondylitis: relationship with disease parameters, psychological status and quality of life. Rheumatol Int 2013;33:1039–45.

12. Baysal O, Durmuş B, Ersoy Y, Altay Z, Senel K, Nas K, *et al.* Relationship between psychological status and disease activity and quality of life in ankylosing spondylitis. Rheumatol Int 2011;31:795–800.

13. Bedaiwi M, Sari I, Thavaneswaran A, Ayearst R, Haroon N, Inman RD. Fatigue in ankylosing spondylitis and nonradiographic axial spondyloarthritis: analysis from a longitudinal observation cohort. J Rheumatol 2015;42:2354–60.

14. Bodur H, Ataman S, Rezvani A, Buğdaycı DS, Cevik R, Birtane M, *et al.* Quality of life and related variables in patients with ankylosing spondylitis. Qual Life Res 2011;20:543–9.

15. Boonen A, Boone C, Albert A, Mielants H. Understanding limitations in at-work productivity in patients with active ankylosing spondylitis: the role of work-related contextual factors. J Rheumatol 2015;42:93–100.

16. Boonen A, Boone C, Albert A, Mielants H. Contextual factors influence work outcomes in employed patients with ankylosing spondylitis starting etanercept: 2-year results from AS@Work. Rheumatology (Oxford) 2018;57:791–7.

17. Brophy S, Davies H, Dennis MS, Cooksey R, Husain MJ, Irvine E, *et al.* Fatigue in ankylosing spondylitis: treatment should focus on pain management. Semin Arthritis Rheum 2013;42:361–7.

18. Cakar E, Dincer U, Kiralp MZ, Taskaynatan MA, Yasar E, Bayman EO, *et al.* Sexual problems in male ankylosing spondylitis patients: relationship with functionality, disease activity, quality of life, and emotional status. Clin Rheumatol 2007;26:1607–13.

19. Cakar E, Taskaynatan MA, Dincer U, Kiralp MZ, Durmus O, Ozgül A. Work disability in ankylosing spondylitis: differences among working and work-disabled patients. Clin Rheumatol 2009;28:1309–14.

20. Carvalho PD, Ruyssen-Witrand A, Marreiros A, Machado PM. Long-term association between disease activity and disability in early axial spondyloarthritis: results from a prospective observational study of inflammatory back pain. Arthritis Care Res (Hoboken) 2022;74:768–75.

21. Cengiz G, Şaş S, Kaplan H, Özsoy S, Çalış M. The influence of alexithymia on disease activity and quality of life in patients with axial spondyloarthritis: a cross-sectional study. Int J Rheum Dis 2023;26:1091–102.

22. Chen CH, Chen HA, Liu CH, Liao HT, Chou CT, Chen CH. Association of obesity with inflammation, disease severity and cardiovascular risk factors among patients with ankylosing spondylitis. Int J Rheum Dis 2020;23:1165–74.

23. Chen CH, Chen HA, Lu CL, Liao HT, Liu CH, Tsai CY, *et al.* Association of cigarette smoking with Chinese ankylosing spondylitis patients in Taiwan: a poor disease outcome in systemic inflammation, functional ability, and physical mobility. Clin Rheumatol 2013;32:659–63.

24. Chen HH, Chen YM, Lai KL, Hsieh TY, Hung WT, Lin CT, *et al.* Gender difference in ASAS HI among patients with ankylosing spondylitis. PLoS One 2020;15:e0235678.

25. Chen MH, Lee MH, Liao HT, Chen WS, Lai CC, Tsai CY. Health-related quality of life outcomes in patients with rheumatoid arthritis and ankylosing spondylitis after tapering biologic treatment. Clin Rheumatol 2018;37:429–38.

26. Chung DXY, Loo YE, Kwan YH, Phang JK, Woon TH, Goh WR, *et al.* Association of anxiety, depression and resilience with overall health and functioning in axial spondyloarthritis (axSpA): a cross-sectional study. BMJ Open 2023;13:e071944.

27. Chung HY, Machado P, van der Heijde D, D'Agostino MA, Dougados M. Smokers in early axial spondyloarthritis have earlier disease onset, more disease activity, inflammation and damage, and poorer function and health-related quality of life: results from the DESIR cohort. Ann Rheum Dis 2012;71:809–16.

28. Connolly D, Fitzpatrick C, O'Shea F. Disease activity, occupational participation, and quality of life for individuals with and without severe fatigue in ankylosing spondylitis. Occup Ther Int 2019;2019:3027280.

29. Cunha RN, Vieira-Sousa E, Khmelinskii N, Ávila-Ribeiro P, Couto M, Seixas MI, *et al.* Sex differences in axial spondyloarthritis: data from a Portuguese spondyloarthritis cohort. ARP Rheumatol 2022;1:42–8.

30. D'Angelo S, Gilio M, D'Attino RM, Gualberti G, Merolla R, di Luzio Paparatti U, *et al.* Observational study on the QUality of life of Italian Axial SpondyloARthritis patients (QUASAR): baseline data. Clin Exp Rheumatol 2019;37:748–55.

31. D'Angelo S, Malavolta N, Scambi C, Salvarani C, Caso F, Tirri E, *et al.* Quality of life and therapeutic management of axial spondyloarthritis patients in Italy: a 12-month prospective observational study. Clin Exp Rheumatol 2021;39:961–9.

32. Demir SE, Rezvani A, Ok S. Assessment of sexual functions in female patients with ankylosing spondylitis compared with healthy controls. Rheumatol Int 2013;33:57–63.

33. Dhakad U, Singh BP, Das SK, Wakhlu A, Kumar P, Srivastava D, *et al.* Sexual dysfunctions and lower urinary tract symptoms in ankylosing spondylitis. Int J Rheum Dis 2015;18:866–72.

34. Dincer U, Cakar E, Kiralp MZ, Bozkanat E, Kilac H, Dursun H. The pulmonary involvement in rheumatic diseases: pulmonary effects of ankylosing spondylitis and its impact on functionality and quality of life. Tohoku J Exp Med 2007;212:423–30.

35. Dincer U, Cakar E, Kiralp MZ, Dursun H. Assessment of sexual dysfunction in male patients with ankylosing spondylitis. Rheumatol Int 2007;27:561–6.

36. Dülger S, Aykurt Karlibel İ, Kasapoğlu Aksoy M, Altan L, Şengören Dikiş Ö, Yildiz T. How does smoking cessation affect disease activity, function loss, and quality of life in smokers with ankylosing spondylitis? J Clin Rheumatol 2019;25:288–96.

37. Durcan L, Wilson F, Conway R, Cunnane G, O'Shea FD. Increased body mass index in ankylosing spondylitis is associated with greater burden of symptoms and poor perceptions of the benefits of exercise. J Rheumatol 2012;39:2310–4.

38. Durmus D, Sarisoy G, Alayli G, Kesmen H, Çetin E, Bilgici A, *et al.* Psychiatric symptoms in ankylosing spondylitis: their relationship with disease activity, functional capacity, pain and fatigue. Compr Psychiatry 2015;62:170–7.

39. Elolemy G, Aboughanima A, Ganeb S, Elziat H. Health-related quality of life in patients with ankylosing spondylitis: relationship with disease-related variables. Curr Rheumatol Rev 2020;16:311–8.

40. Espahbodi S, Bassett P, Cavill C, Freeth M, Hole J, Sengupta R. Fatigue contributes to work productivity impairment in patients with axial spondyloarthritis: a cross-sectional UK study. Clin Exp Rheumatol 2017;35:571–8.

41. Essers I, Boonen A, Busch M, van der Heijde D, Keszei AP, Landewé R, *et al.* Fluctuations in patient reported disease activity, pain and global being in patients with ankylosing spondylitis. Rheumatology (Oxford) 2016;55:2014–22.

42. Fernández-Carballido C, Martín-Martínez MA, García-Gómez C, Castañeda S, González-Juanatey C, Sánchez-Alonso F, *et al.* Impact of comorbidity on physical function in patients with ankylosing spondylitis and psoriatic arthritis attending rheumatology clinics: results from a cross-sectional study. Arthritis Care Res (Hoboken) 2020;72:822–8.

43. Fernández-Carballido C, Navarro-Compán V, Castillo-Gallego C, Castro-Villegas MC, Collantes-Estévez E, de Miguel E. Disease activity as a major determinant of quality of life and physical function in patients with early axial spondyloarthritis. Arthritis Care Res (Hoboken) 2017;69:150–5.

44. Fongen C, Sveaas SH, Dagfinrud H. Barriers and facilitators for being physically active in patients with ankylosing spondylitis: a cross-sectional comparative study. Musculoskeletal Care 2015;13:76–83.

45. Forejtová S, Mann H, Stolfa J, Vedral K, Fenclová I, Némethová D, *et al.* Factors influencing health status and disability of patients with ankylosing spondylitis in the Czech Republic. Clin Rheumatol 2008;27:1005–13.

46. Frede N, Rieger E, Lorenzetti R, Venhoff AC, Kanne AM, Finzel S, *et al.* Sleep behaviour differs in women and men with psoriatic arthritis and axial spondyloarthritis with impact on quality of life and depressive symptoms. RMD Open 2023;9:e002912.

47. Garrido-Cumbrera M, Bundy C, Navarro-Compán V, Makri S, Sanz-Gómez S, Christen L, *et al.* Patient-reported impact of axial spondyloarthritis on working life: results from the European map of axial spondyloarthritis survey. Arthritis Care Res (Hoboken) 2021;73:1826–33.

48. Garrido-Cumbrera M, Collantes-Estevez E, Navarro-Compán V, Zarco-Montejo P, Sastre C, Correa-Fernández J, *et al.* Understanding the disease burden of unemployed patients with axial spondyloarthritis: results from the Spanish Atlas 2017. J Rheumatol 2022;49:373–9.

49. Garrido-Cumbrera M, Delgado-Domínguez CJ, Gálvez-Ruiz D, Mur CB, Navarro-Compán V. The effect of axial spondyloarthritis on mental health: results from the Atlas. J Rheumatol 2019;46:1284–9.

50. Garrido-Cumbrera M, Gálvez-Ruiz D, Delgado-Domínguez CJ, Poddubnyy D, Navarro-Compán V, Christen L, *et al.* Impact of axial spondyloarthritis on mental health in Europe: results from the EMAS study. RMD Open 2021;7:e001769.

51. Gok K, Cengiz G, Erol K, Ozgocmen S. Neuropathic pain component in axial spondyloarthritis and the influence on disease burden. J Clin Rheumatol 2018;24:324–7.

52. Gossec L, Dougados M, D'Agostino MA, Fautrel B. Fatigue in early axial spondyloarthritis. Results from the French DESIR cohort. Joint Bone Spine 2016;83:427–31.

53. Gözüküçük M, Türkyilmaz E, Küçükşahin O, Erten Ş, Üstün Y, Yavuz AF. Effects of ankylosing spondylitis and non-radiographic axial spondyloarthropathy on female sexual functions. Clin Exp Rheumatol 2022;40:967–74.

54. Günaydin R, Göksel Karatepe A, Ceşmeli N, Kaya T. Fatigue in patients with ankylosing spondylitis: relationships with disease-specific variables, depression, and sleep disturbance. Clin Rheumatol 2009;28:1045–51.

55. Hakkou J, Rostom S, Aissaoui N, Berrada KR, Abouqal R, Bahiri R, *et al.* Psychological status in Moroccan patients with ankylosing spondylitis and its relationships with disease parameters and quality of life. J Clin Rheumatol 2011;17:424–8.

56. Hakkou J, Rostom S, Mengat M, Aissaoui N, Bahiri R, Hajjaj-Hassouni N. Sleep disturbance in Moroccan patients with ankylosing spondylitis: prevalence and relationships with disease-specific variables, psychological status and quality of life. Rheumatol Int 2013;33:285–90.

57. Haywood KL, Packham JC, Jordan KP. Assessing fatigue in ankylosing spondylitis: the importance of frequency and severity. Rheumatology (Oxford) 2014;53:552–6.

58. Healey EL, Haywood KL, Jordan KP, Garratt A, Packham JC. Impact of ankylosing spondylitis on work in patients across the UK. Scand J Rheumatol 2011;40:34–40.

59. Healey EL, Haywood KL, Jordan KP, Garratt AM, Ryan S, Packham JC. Ankylosing spondylitis and its impact on sexual relationships. Rheumatology (Oxford) 2009;48:1378–81.

60. Heiberg T, Lie E, van der Heijde D, Kvien TK. Sleep problems are of higher priority for improvement for patients with ankylosing spondylitis than for patients with other inflammatory arthropathies. Ann Rheum Dis 2011;70:872–3.

61. Huang JC, Qian BP, Qiu Y, Wang B, Yu Y, Zhu ZZ, *et al.* Quality of life and correlation with clinical and radiographic variables in patients with ankylosing spondylitis: a retrospective case series study. BMC Musculoskelet Disord 2017;18:352.

62. Hwang MC, Lee MJ, Gensler LS, Ward MM, Brown MA, Eisen S, *et al.* Longitudinal associations between depressive symptoms and clinical factors in ankylosing spondylitis patients: analysis from an observational cohort. Rheumatol Int 2020;40:1053–61.

63. Ibn Yacoub Y, Amine B, Laatiris A, Abouqal R, Hajjaj-Hassouni N. Assessment of fatigue in Moroccan patients with ankylosing spondylitis. Clin Rheumatol 2010;29:1295–9.

64. Ibn Yacoub Y, Amine B, Laatiris A, Abouqal R, Hajjaj-Hassouni N. Health-related quality of life in Moroccan patients with ankylosing spondylitis. Clin Rheumatol 2011;30:673–7.

65. Jiang Y, Yang M, Lv Q, Qi J, Lin Z, Liao Z, *et al.* Prevalence of psychological disorders, sleep disturbance and stressful life events and their relationships with disease parameters in Chinese patients with ankylosing spondylitis. Clin Rheumatol 2018;37:407–14.

66. Jiang Y, Yang M, Wu H, Song H, Zhan F, Liu S, *et al.* The relationship between disease activity measured by the BASDAI and psychological status, stressful life events, and sleep quality in ankylosing spondylitis. Clin Rheumatol 2015;34:503–10.

67. Kankaya H, Özer S, Yan G, Erden B, Şaraldı F, Bebek S. The quality of life in Muslim patients with ankylosing spondylitis in Türkiye. J Transcult Nurs 2023;34:195–200.

68. Karatekin BD, Icagasioglu A, Oguz FM, Bayindir SN, Pasin O. Trajectory of anxiety, depression and quality of life in ankylosing spondylitis: a descriptive study. J Coll Physicians Surg Pak 2023;33:314–8.

69. Kedra J, Claudepierre P, Flipo RM, Garrido-Cumbrera M, Alliot-Launois F, Desfleurs E, *et al.* Impact of axial spondyloarthritis on quality of life: results from the European Map of Axial Spondyloarthritis (EMAS) study in France. J Rheumatol 2022;49:1176–8.

70. Kieskamp SC, Paap D, Carbo MJG, Wink F, Bos R, Bootsma H, *et al.* Central sensitization has major impact on quality of life in patients with axial spondyloarthritis. Semin Arthritis Rheum 2022;52:151933.

71. Kiltz U, Hoeper K, Hammel L, Lieb S, Hähle A, Meyer-Olson D. Work participation in patients with axial spondyloarthritis: high prevalence of negative workplace experiences and long-term work impairment. RMD Open 2023;9:e002663.

72. Korotaeva T, Dina O, Holdsworth E, Fallon L, Milligan G, Meakin S, *et al.* Investigating diagnosis, treatment, and burden of disease in patients with ankylosing spondylitis in Central Eastern Europe and the United States: a real-world study. Clin Rheumatol 2021;40:4915–26.

73. Kydd AS, Chen JS, Makovey J, Chand V, Henderson L, Buchbinder R, *et al.* Smoking did not modify the effects of anti-TNF treatment on health-related quality of life among Australian ankylosing spondylitis patients. Rheumatology (Oxford) 2015;54:310–7.

74. Landewé R, Dougados M, Mielants H, van der Tempel H, van der Heijde D. Physical function in ankylosing spondylitis is independently determined by both disease activity and radiographic damage of the spine. Ann Rheum Dis 2009;68:863–7.

75. Law L, Beckman Rehnman J, Deminger A, Klingberg E, Jacobsson LTH, Forsblad-d'Elia H. Factors related to health-related quality of life in ankylosing spondylitis, overall and stratified by sex. Arthritis Res Ther 2018;20:284.

76. Li Y, Zhang S, Zhu J, Du X, Huang F. Sleep disturbances are associated with increased pain, disease activity, depression, and anxiety in ankylosing spondylitis: a case-control study. Arthritis Res Ther 2012;14:R215.

77. Lu MC, Huang KY, Tung CH, Hsu BB, Wu CH, Koo M, *et al.* Factors associated with disease-specific quality of life in Taiwanese patients with ankylosing spondylitis: a cross-sectional study. BMJ Open 2019;9:e028966.

78. Machado P, Landewé R, Braun J, Hermann KG, Baraliakos X, Baker D, *et al.* A stratified model for health outcomes in ankylosing spondylitis. Ann Rheum Dis 2011;70:1758–64.

79. Magrey M, Walsh JA, Flierl S, Howard RA, Calheiros RC, Wei D, *et al.* The international map of axial spondyloarthritis survey: a US patient perspective on diagnosis and burden of disease. ACR Open Rheumatol 2023;5:264–76.

80. Maguire S, Wilson F, Gallagher P, O'Shea F. The toll of unemployment in axial spondyloarthropathy: high prevalence and negative impact on outcomes captured in a national registry. Scand J Rheumatol 2022;51:300–3.

81. Maguire S, Wilson F, Gallagher P, O'Shea F. Central obesity in axial spondyloarthritis: the missing link to understanding worse outcomes in women? J Rheumatol 2022;49:577–84.

82. Maksymowych WP, Gooch KL, Wong RL, Kupper H, van der Heijde D. Impact of age, sex, physical function, health-related quality of life, and treatment with adalimumab on work status and work productivity of patients with ankylosing spondylitis. J Rheumatol 2010;37:385–92.

83. Mattey DL, Dawson SR, Healey EL, Packham JC. Relationship between smoking and patient-reported measures of disease outcome in ankylosing spondylitis. J Rheumatol 2011;38:2608–15.

84. Mease PJ, McLean RR, Dube B, Liu M, Rebello S, Glynn M, *et al.* Comparison of men and women with axial spondyloarthritis in the US-based corrona psoriatic arthritis/spondyloarthritis registry. J Rheumatol 2021;48:1528–36.

85. Mlcoch T, Sedova L, Stolfa J, Urbanova M, Suchy D, Smrzova A, *et al.* Mapping the relationship between clinical and quality-of-life outcomes in patients with ankylosing spondylitis. Expert Rev Pharmacoecon Outcomes Res 2017;17:203–11.

86. Morton L, Macfarlane GJ, Jones G, Walker-Bone K, Hollick R. Driving difficulties in patients with axial spondyloarthritis: results from the Scotland Registry for Ankylosing Spondylitis. Arthritis Care Res (Hoboken) 2022;74:1541–9.

87. Mustur D, Vesović-Potić V, Stanisavljević D, Ille T, Ille M. Assessment of functional disability and quality of life in patients with ankylosing spondylitis. Srp Arh Celok Lek 2009;137:524–8.

88. Nam B, Koo BS, Nam SW, Shin JH, Song Y, Cho SK, *et al.* Gender differences in factors associated with low quality of life and depression in Korean patients with ankylosing spondylitis. Qual Life Res 2021;30:2299–310.

89. Nie A, Wang C, Song Y, Xie X, Yang H, Chen H. Prevalence and factors associated with disturbed sleep in outpatients with ankylosing spondylitis. Clin Rheumatol 2018;37:2161–8.

90. Nikiphorou E, Boonen A, Fautrel B, Richette P, Landewé R, van der Heijde D, *et al.* How do clinical and socioeconomic factors impact on work disability in early axial spondyloarthritis? Five-year data from the DESIR cohort. Rheumatology (Oxford) 2022;61:2034–42.

91. Nikiphorou E, Ramiro S, van der Heijde D, Norton S, Moltó A, Dougados M, *et al.* Association of comorbidities in spondyloarthritis with poor function, work disability, and quality of life: results from the assessment of SpondyloArthritis international Society Comorbidities in Spondyloarthritis study. Arthritis Care Res (Hoboken) 2018;70(8):1257–62.

92. O'Dwyer T, O'Shea F, Wilson F. Decreased physical activity and cardiorespiratory fitness in adults with ankylosing spondylitis: a cross-sectional controlled study. Rheumatol Int 2015;35:1863–72.

93. O'Dwyer T, O'Shea F, Wilson F. Decreased health-related physical fitness in adults with ankylosing spondylitis: a cross-sectional controlled study. Physiotherapy 2016;102:202–9.

94. Öğüt TS, Erbasan F, Terzioğlu ME, Aslan B, Çelik E, Yazisiz V. Neuropathic pain in axial spondyloarthropathy is underdiagnosed and a confounding factor in biologic drug-switching decision: a cross-sectional study. Clin Rheumatol 2023;42:1275–84.

95. Öksüz E, Cinar FI, Cinar M, Tekgoz E, Yilmaz S. Assessment of the effects of loneliness, perceived social support, and depression on medication adherence in patients with ankylosing spondylitis. Perspect Psychiatr Care 2021;57:517–23.

96. Omar M, Ben-Shabat N, Tsur AM, Cohen AD, Watad A, Amital H, *et al.* The association between ankylosing spondylitis and psychiatric disorders: insights from a population based cross-sectional database. J Affect Disord 2023;323:788–92.

97. Ortancil O, Konuk N, May H, Sanli A, Ozturk D, Ankarali H. Psychological status and patient-assessed health instruments in ankylosing spondylitis. J Clin Rheumatol 2010;16:313–6.

98. Ozdemir O. Quality of life in patients with ankylosing spondylitis: relationships with spinal mobility, disease activity and functional status. Rheumatol Int 2011;31:605–10.

99. Park JS, Jang HD, Hong JY, Park YS, Han K, Suh SW, *et al.* Impact of ankylosing spondylitis on depression: a nationwide cohort study. Sci Rep 2019;9:6736.

100. Poddubnyy D, Haibel H, Listing J, Märker-Hermann E, Zeidler H, Braun J, *et al.* Baseline radiographic damage, elevated acute-phase reactant levels, and cigarette smoking status predict spinal radiographic progression in early axial spondylarthritis. Arthritis Rheum 2012;64:1388–98.

101. Poddubnyy D, Haibel H, Listing J, Märker-Hermann E, Zeidler H, Braun J, *et al.* Cigarette smoking has a dose-dependent impact on progression of structural damage in the spine in patients with axial spondyloarthritis: results from the GErman SPondyloarthritis Inception Cohort (GESPIC). Ann Rheum Dis 2013;72:1430–2.

102. Rafia R, Ara R, Packham J, Haywood KL, Healey E. Healthcare costs and productivity losses directly attributable to ankylosing spondylitis. Clin Exp Rheumatol 2012;30:246–53.

103. Redeker I, Hoffmann F, Callhoff J, Haibel H, Sieper J, Zink A, *et al.* Determinants of psychological well-being in axial spondyloarthritis: an analysis based on linked claims and patient-reported survey data. Ann Rheum Dis 2018;77:1017–24.

104. Rezvani A, Bodur H, Ataman S, Kaya T, Buğdaycı DS, Demir SE, *et al.* Correlations among enthesitis, clinical, radiographic and quality of life parameters in patients with ankylosing spondylitis. Mod Rheumatol 2014;24:651–6.

105. Rohde G, Berg KH, Pripp AH, Prøven A, Haugeberg G. No deterioration in health-related quality of life in patients with axial spondyloarthritis followed for 5 years in ordinary outpatient clinics in the biological treatment era. Qual Life Res 2020;29:99–107.

106. Rosas J, Llinares-Tello F, Senabre-Gallego JM, Barber-Vallés X, Santos-Soler G, Salas-Heredia E, *et al.* Obesity decreases clinical efficacy and levels of adalimumab in patients with ankylosing spondylitis. Clin Exp Rheumatol 2017;35:145–8.

107. Rostom S, Mengat M, Mawani N, Jinane H, Bahiri R, Hajjaj-Hassouni N. Sexual activity in Moroccan men with ankylosing spondylitis. Rheumatol Int 2013;33:1469–74.

108. Sağ S, Nas K, Sağ MS, Tekeoğlu İ, Kamanlı A. Relationship of work disability between the disease activity, depression and quality of life in patients with ankylosing spondylitis. J Back Musculoskelet Rehabil 2018;31:499–505.

109. Sakellariou GT, Anastasilakis AD, Kenanidis E, Potoupnis M, Tsiridis E, Savvidis M, *et al.* The effect of smoking on clinical and radiographic variables, and acute phase reactants in patients with ankylosing spondylitis. Rheumatol Int 2015;35:2109–14.

110. Santana T, Skare T, Delboni VS, Simione J, Campos APB, Nisihara R. Erectile dysfunction in ankylosing spondylitis patients. Int Braz J Urol 2017;43:730–5.

111. Sariyildiz MA, Batmaz I, Dilek B, Inanir A, Bez Y, Tahtasiz M, *et al.* Relationship of the sexual functions with the clinical parameters, radiological scores and the quality of life in male patients with ankylosing spondylitis. Rheumatol Int 2013;33:623–9.

112. Sariyildiz MA, Batmaz I, Inanir A, Dilek B, Bozkurt M, Bez Y, *et al.* The impact of ankylosing spondylitis on female sexual functions. Int J Impot Res 2013;25:104–8.

113. Schmalz G, Douglas D, Douglas D, Patschan S, Patschan D, Müller GA, *et al.* Oral health-related quality of life is associated with disease specific parameters in patients with ankylosing spondylitis. Clin Oral Investig 2018;22:2889–96.

114. Schneeberger EE, Marengo MF, Dal Pra F, Maldonado Cocco JA, Citera G. Fatigue assessment and its impact in the quality of life of patients with ankylosing spondylitis. Clin Rheumatol 2015;34:497–501.

115. Shahlaee A, Mahmoudi M, Nicknam MH, Farhadi E, Fallahi S, Jamshidi AR. Gender differences in Iranian patients with ankylosing spondylitis. Clin Rheumatol 2015;34:285–93.

116. Shen B, Zhang A, Liu J, Da Z, Xu X, Gu Z. A primary analysis of sexual problems in Chinese patients with ankylosing spondylitis. Rheumatol Int 2013;33:1429–35.

117. Shen B, Zhang A, Liu J, Da Z, Xu X, Liu H, *et al.* Body image disturbance and quality of life in Chinese patients with ankylosing spondylitis. Psychol Psychother 2014;87:324–37.

118. Shen CC, Hu LY, Yang AC, Kuo BI, Chiang YY, Tsai SJ. Risk of psychiatric disorders following ankylosing spondylitis: a nationwide population-based retrospective cohort study. J Rheumatol 2016;43:625–31.

119. Sieper J, Holbrook T, Black CM, Wood R, Hu X, Kachroo S. Burden of illness associated with non-radiographic axial spondyloarthritis: a multiperspective European cross-sectional observational study. Clin Exp Rheumatol 2016;34:975–83.

120. Slobodin G, Reyhan I, Avshovich N, Balbir-Gurman A, Boulman N, Elias M, *et al.* Recently diagnosed axial spondyloarthritis: gender differences and factors related to delay in diagnosis. Clin Rheumatol 2011;30:1075–80.

121. Stebbings SM, Treharne GJ, Jenks K, Highton J. Fatigue in patients with spondyloarthritis associates with disease activity, quality of life and inflammatory bowel symptoms. Clin Rheumatol 2014;33:1467–74.

122. Strand V, Deodhar A, Alten R, Sullivan E, Blackburn S, Tian H, *et al.* Pain and fatigue in patients with ankylosing spondylitis treated with tumor necrosis factor inhibitors: multinational real-world findings. J Clin Rheumatol 2021;27:e446-55.

123. Swinnen TW, Westhovens R, Dankaerts W, de Vlam K. Widespread pain in axial spondyloarthritis: clinical importance and gender differences. Arthritis Res Ther 2018;20:156.

124. Tam LS, Chan KY, Li EK. The influence of illness and variables associated with functional limitations in Chinese patients with ankylosing spondylitis. J Rheumatol 2007;34:1032–9.

125. Tarhan F, Tarhan H, Karaoğullarından U, Can E, Divrik T, Zorlu F. Premature ejaculation in patients with ankylosing spondylitis. Int J Androl 2012;35:74–8.

126. Taser B, Ayhan FF, Borman P. The importance of quality of life for work outcomes in patients with ankylosing spondylitis - a cross-sectional study. Acta Reumatol Port 2017;42:300–9.

127. Tu L, Rai JC, Cao S, Lin Z, Hu Z, Gu J. Costs and work limitation of patients with ankylosing spondylitis in China. Clin Exp Rheumatol 2014;32:661–6.

128. Turan Y, Duruöz MT, Bal S, Guvenc A, Cerrahoglu L, Gurgan A. Assessment of fatigue in patients with ankylosing spondylitis. Rheumatol Int 2007;27:847–52.

129. Turan Y, Duruöz MT, Cerrahoglu L. Quality of life in patients with ankylosing spondylitis: a pilot study. Rheumatol Int 2007;27:895–9.

130. Tymms K, Butcher BE, Sletten TL, Smith T, O'Sullivan C, Littlejohn G, *et al.* Prevalence of sleep disturbance and the association between poor disease control in people with ankylosing spondylitis within the Australian clinical setting (ASLEEP study): a real-world observational study using the OPAL dataset. Clin Rheumatol 2022;41:1105–14.

131. Uludag M, Unalan H, Tuzun S, Kocabasoglu N, Aydin FY, Palamar D, *et al.* Assessment of quality of life and depression in spouses of patients with ankylosing spondylitis. Rheumatol Int 2012;32:3511–6.

132. Ulus Y, Akyol Y, Bilgici A, Kuru O. Association of work instability with fatigue and emotional status in patients with ankylosing spondylitis: comparison with healthy controls. Clin Rheumatol 2019;38:1017–24.

133. Urkmez B, Keskin Y. Relationship between sleep quality and physical activity level in patients with ankylosing spondylitis. Mod Rheumatol 2020;30:1053–9.

134. van der Heijde D, Joshi A, Pangan AL, Chen N, Betts K, Mittal M, *et al.* ASAS40 and ASDAS clinical responses in the ABILITY-1 clinical trial translate to meaningful improvements in physical function, health-related quality of life and work productivity in patients with non-radiographic axial spondyloarthritis. Rheumatology (Oxford) 2016;55:80–8.

135. van der Horst-Bruinsma IE, Zack DJ, Szumski A, Koenig AS. Female patients with ankylosing spondylitis: analysis of the impact of gender across treatment studies. Ann Rheum Dis 2013;72:1221–4.

136. van Genderen S, Plasqui G, Landewé R, Lacaille D, Arends S, van Gaalen F, *et al.* Social role participation in patients with ankylosing spondylitis: a cross-sectional comparison with population controls. Arthritis Care Res (Hoboken) 2016;68:1899–905.

137. van Genderen S, Plasqui G, van der Heijde D, van Gaalen F, Heuft L, Luime J, *et al.* Social role participation and satisfaction with life: a study among patients with ankylosing spondylitis and population controls. Arthritis Care Res (Hoboken) 2018;70:600–7.

138. van Genderen S, van den Borne C, Geusens P, van der Linden S, Boonen A, Plasqui G. Physical functioning in patients with ankylosing spondylitis: comparing approaches of experienced ability with self-reported and objectively measured physical activity. J Clin Rheumatol 2014;20:133–7.

139. van Lunteren M, Ez-Zaitouni Z, de Koning A, Dagfinrud H, Ramonda R, Jacobsson L, *et al.* In early axial spondyloarthritis, increasing disease activity is associated with worsening of health-related quality of life over time. J Rheumatol 2018;45:779–84.

140. van Lunteren M, Ez-Zaitouni Z, Fongen C, Landewé R, Ramonda R, van der Heijde D, *et al.* Disease activity decrease is associated with improvement in work productivity over 1 year in early axial spondyloarthritis (SPondyloArthritis Caught Early cohort). Rheumatology (Oxford) 2017;56:2222–8.

141. Vergne-Salle P, Salle L, Fressinaud-Marie AC, Descamps-Deplas A, Montestruc F, Bonnet C, *et al.* Diet and disease activity in patients with axial spondyloarthritis: SpondyloArthritis and NUTrition Study (SANUT). Nutrients 2022;14:4730.

142. Vesović-Potić V, Mustur D, Stanisavljević D, Ille T, Ille M. Relationship between spinal mobility measures and quality of life in patients with ankylosing spondylitis. Rheumatol Int 2009;29:879–84.

143. Vitturi BK, Suriano ES, Pereira de Sousa AB, Torigoe DY. Cognitive impairment in patients with ankylosing spondylitis. Can J Neurol Sci 2020;47:219–25.

144. Wadeley A, Clarke E, Leverment S, Sengupta R. Sleep in ankylosing spondylitis and non-radiographic axial spondyloarthritis: associations with disease activity, gender and mood. Clin Rheumatol 2018;37:1045–52.

145. Walsh JA, Song X, Kim G, Park Y. Evaluation of the comorbidity burden in patients with ankylosing spondylitis using a large US administrative claims data set. Clin Rheumatol 2018;37:1869–78.

146. Ward MM, Reveille JD, Learch TJ, Davis JC, Jr., Weisman MH. Impact of ankylosing spondylitis on work and family life: comparisons with the US population. Arthritis Rheum 2008;59:497–503.

147. Wu JJ, Penfold RB, Primatesta P, Fox TK, Stewart C, Reddy SP, *et al.* The risk of depression, suicidal ideation and suicide attempt in patients with psoriasis, psoriatic arthritis or ankylosing spondylitis. J Eur Acad Dermatol Venereol 2017;31:1168–75.

148. Yılmaz O, Tutoğlu A, Garip Y, Ozcan E, Bodur H. Health-related quality of life in Turkish patients with ankylosing spondylitis: impact of peripheral involvement on quality of life in terms of disease activity, functional status, severity of pain, and social and emotional functioning. Rheumatol Int 2013;33:1159–63.

149. Yüce E, Şentürk E, Sağaltıcı E, Şentürk İ A, Aytekin E. Sleep quality and depression in patients with ankylosing spondylitis and their associations with clinical parameters: a cross-sectional, case-control study. Agri 2023;35:1–9.

150. Žagar I, Delimar V, Čota S, Špoljarić Carević S, Kovač Durmiš K, Laktašić Žerjavić N, *et al.* The impact of disease activity on quality of life, fatigue, functional status and physical activity in patients with ankylosing spondylitis. Psychiatr Danub 2021;33(Suppl 4):1278–83.

151. Zhang H, Wan W, Liu J, Dai S, Zou Y, Qian Q, *et al.* Smoking quantity determines disease activity and function in Chinese patients with ankylosing spondylitis. Clin Rheumatol 2018;37:1605–16.

152. Zhou W, Guo J, He M, Li J, Chen Y, Liu J, *et al.* Fatigue and contributing factors in Chinese patients with ankylosing spondylitis. Clin Rheumatol 2020;39:2337–44.

153. Zviahina OV, Shevchuk SV, Kuvikova IP, Segeda IS. Anemia in patients with ankylosing spondylitis, association with the activity of the inflammatory process and the severity of the disease. Wiad Lek 2020;73:715–21.

154. Hwang MC, Rozycki M, Kauffman D, Arndt T, Yi E, Weisman MH. Does gender impact a diagnosis of ankylosing spondylitis? ACR Open Rheumatol 2022;4:540–6.

155. Mease PJ, Liu M, Rebello S, Hua W, McLean RR, Yi E, *et al.* Characterization of patients with axial spondyloarthritis by enthesitis presence: data from the Corrona Psoriatic Arthritis/Spondyloarthritis Registry. ACR Open Rheumatol 2020;2:449–56.

156. Rausch Osthoff AK, Nast I, Niedermann K. Understanding beliefs related to physical activity in people living with axial spondyloarthritis: a theory-informed qualitative study. BMC Rheumatol 2022;6:40.

157. Park JY, Howren AM, Zusman EZ, Esdaile JM, De Vera MA. The incidence of depression and anxiety in patients with ankylosing spondylitis: a systematic review and meta-analysis. BMC Rheumatol 2020;4:12.

158. Phang JK, Khor AYK, Kwan YH, Ng CT, Fong W. Physical activity in patients with axial spondyloarthritis in a multi-ethnic south-east Asian country. BMC Rheumatol 2021;5:38.

159. Tang H, Singh BSM, Fong W. Prevalence and factors associated with fatigue in patients with axial spondyloarthritis: a systematic review and meta-analysis. Rheumatol Adv Pract 2023;7:rkad084.

160. Wilson N, Liu J, Adamjee Q, Di Giorgio S, Steer S, Hutton J, *et al.* Exploring the emotional impact of axial spondyloarthritis: a systematic review and thematic synthesis of qualitative studies and a review of social media. BMC Rheumatol 2023;7:26.

161. Chang YS, Shen HM, Lin LF, Peng HY, Chen PC, Zeng YH, *et al.* Attitudes and barriers to exercise in Taiwanese ankylosing spondylitis patients. Int J Rheum Dis 2018;21:SU013.

162. De Silva S, Abeysinghe D. Health related quality of life of patients with axial spondyloarthropathy and associated factors at a tertiary care in Sri Lanka. Poster Presentations 2021. Int J Rheum Dis 24:274.

163. Dong C, Wang J, Wu X, Li Z, Ji J, Fu T, *et al.* Syndesmophyte affect functional status, spinal mobility, pain, disability, and health related quality of life in patients with ankylosing spondylitis [abstract]. Int J Rheum Dis 2018:SU160.

164. Ediboglu E, Solmaz D, Ece Oz H, Kabadayı G, Cinaklı H, Otman Akat E, *et al.* Work disability and predictors of poor work outcome in patients with axial spondyloarthritis [abstract]. Arthritis Rheumatol 2020;72(Suppl 10):0165.

165. Fongen C, Solveig Dagfinrud H, Bilberg A, Sveaas S. POS0211 HPR reduced sleep quality is highly prevalent and associated with physical function and cardiorespiratory fitness in patients with axial spondyloarthritis. Ann Rheum Dis 2023;82(Suppl 1):332.

166. Garrido-Cumbrera M, Bundy C, Navarro-Compán V, Christen L, Mahapatra R, Makri S, *et al.* POS0989 Factors associated with inability to work and disability in patients with axial spondyloarthritis. Results from the European Map of Axial Spondyloarthritis (EMAS). Ann Rheum Dis 2021;80(Suppl 1):762–3.

167. Garrido-Cumbrera M, Bundy C, Poddubnyy D, Makri S, Mahapatra R, Sanz-Gómez S, *et al.* OP0081 The impact of axial spondyloarthritis on patients' sexual life: results from the European Map of Axial Spondyloarthritis (EMAS). Ann Rheum Dis 2020;79(Suppl 1):54–5.

168. Garrido-Cumbrera M, Collantes-Estévez E, Navarro-Compán V, Zarco P, Sastre C, Sanz-Gomez S, *et al.* Patient-reported impact of axial spondyloarthritis on working life. Results from the Spanish Atlas 2017 [abstract]. Arthritis Rheumatol 2020;72(Suppl 10):1866.

169. Garrido-Cumbrera M, Gálvez-Ruiz D, Zarco P, Braçe O, Navarro-Compán V. FRI0206 Gender differences in patients with axial spondyloarthritis: results from the Atlas-2017. Ann Rheum Dis 2018;77(Suppl 2):644.

170. Garrido-Cumbrera M, Navarro-Compán V, Bundy C, Christen L, Mahapatra R, Makri S, *et al.* POS0988 Factors associated with pain intensity in axial spondyloarthritis. Results from the European Map of Axial Spondyloarthritis (EMAS). Ann Rheum Dis 2021;80(Suppl 1):762.

171. Garrido-Cumbrera M, Navarro-Compán V, Bundy C, Mahapatra R, Makri S, Sanz-Gómez S, *et al.* SAT0374 Onset of axial spondyloarthritis repercussions on patients' social and family life: results from the European Map of Axial Spondyloarthritis (EMAS). Ann Rheum Dis 2020;79(Suppl 1):1134–5.

172. Garrido-Cumbrera M, Navarro-Compán V, Bundy C, Makri S, Christen L, Correa-Fernández J, *et al.* Why is it so difficult for AxSpA patients to find a job? Results from the European Map of Axial Spondyloarthritis (EMAS) [abstract]. Arthritis Rheumatol 2021;73:0366.

173. Garrido-Cumbrera M, Navarro-Compán V, Christen L, Bundy C, Mahapatra R, Makri S, *et al.* POS0961 Prevalence and associated factors of sleep disorders in patients with axial spondyloarthritis. Results from the European Map of Axial Spondyloarthritis (EMAS). Ann Rheum Dis 2021;80(Suppl 1):745.

174. Garrido-Cumbrera M, Navarro-Compán V, Christen L, Bundy C, Mahapatra R, Makri S, *et al.* PANLAR Abstracts 2021. Prevalence and associated factors of sleep disorders in patients with axial spondyloarthritis. Results from the European Map of Axial Spondyloarthritis (EMAS). J Clin Rheumatol 2021;27(5S):S1–S146.

175. Gossec L, Garrido-Cumbrera M, Poddubnyy D, Galvez-Ruiz D, Bundy C, Delgado Dominguez CJ, *et al.* Diagnostic delay and associated factors in axial spondyloarthritis across Europe. Results from the European Map of Axial Spondyloarthritis survey [abstract]. Arthritis Rheumatol 2018;70:638.

176. Ibanez S, van Bentum R, Valenzuela O, van der Horst-Bruinsma I. Axial spondyloarthritis patients report important impairments in daily life and work ability – a web survey in 472 patients [abstract]. Arthritis Rheumatol 2019;71:430.

177. Kabir T, Islam N, Ahsan M. Anxiety and depression in axial spondyloarthritis patients: a pilot study from Bangladesh. Poster Presentations 2021. Int J Rheum Dis 2021;24:275.

178. Kleinert S, Schuch F, Rapp P, Ronneberger M, Wendler J, Sternad P, *et al.* Impairment of memory in axial spondyloarthritis? [abstract]. Arthritis Rheumatol 2021;73:1317.

179. Kwan YH, Fong W, Ying LY, Lee LN, Seng TC, Malhotra R, *et al.* 00010 Are peripheral arthritis, enthesitis or dactylitis associated with poorer quality of life in axial spondyloarthritis patients? A 1-year cohort study. Oral Presentation Abstracts 2018. SingHealth Duke-NUS Scientific Congress. 2018.

180. Lim W, Fong W, Heng Kwan Y, Leung YY. Exploring the prevalence and factors associated with fatigue in axial spondyloarthritis in a multiethnic Asian cohort in Singapore [abstract]. Arthritis Rheumatol 2020;72(Suppl 10):1315.

181. Liu LC, Lu MC. Factors associated with disease-specific quality of life in Taiwanese patients with ankylosing spondylitis. 2018 Poster Abstracts. Int J Rheum Dis 2018;21.

182. Liu X, Wang Y, Chen W, Mo S, Ji X, Huang F. Work productivity is associated with disease activity and functional ability in Chinese patients with axial spondyloarthritis using a smart-phone management system: a prospective cohort study [abstract]. Arthritis Rheumatol 2019;71(Suppl 10):418.

183. López-Medina C, Ruiz D, Puche Larrubia MÁ, Ladehesa Pineda ML, Ábalos-Aguilera MC, Pilar FU, *et al.* POS0662 The socioeconomic profile of patients with radiographic axial spondyloarthritis is associated with the severity of the disease and with the permanent disability. A cluster analysis in a national Spanish registry. Ann Rheum Dis 2023;82(Suppl 1):610–1.

184. Maguire S, Fiona W, Gallagher P, O'Shea F. Identifying predictors of unemployment in axial spondyloarthropathy: data from the Ankylosing Spondylitis Registry of Ireland [abstract]. Arthritis Rheumatol 2021;73(Suppl 9):0357.

185. Maguire S, Gallagher P, O'Shea F. High frequency of complications in axial spondyloarthropathy pregnancies: emerging data from the Ankylosing Spondylitis Registry of Ireland [abstract]. Arthritis Rheumatol 2021;73(Suppl 9):0369.

186. Maguire S, Gallagher P, O'Shea F. Undiagnosed depression in axial spondyloarthropathy and the negative impact on patient outcomes: results of a screening study [abstract]. Arthritis Rheumatol 2021;73(Suppl 9):1305.

187. Poddubnyy D, Sieper J, Akar S, Muñoz-Fernández S, Haibel H, Ganz F, *et al.* Disease course and disease burden in patients with axial spondyloarthritis: results from 5-year multicountry prospective observational study [abstract]. Arthritis Rheumatol 2021;73(Suppl 9):0363.

188. Polanco Mora T, Peralta De Heyaime JS, Cornelio Vasquez AA, Cruz Y, Valdez Lorie T, Rodriguez Bautista E, *et al.* PANLAR Abstracts 2021. ABS-1263 Assessment of fatigue in ankylosing spondylitis at Padre Billini Hospital, Dominican Republic. J Clin Rheumatol 27(5S):S127.

189. Redeker I, Hoffmann F, Callhoff J, Haibel H, Sieper J, Zink A, *et al.* FRI0198 Which factors influence psychological well-being of patients with axial spondyloarthritis? – Data from a cross-sectional survey linked to insurance claims. Ann Rheum Dis 2018;77(Suppl 2):640.

190. Regierer A, Weiß A, Baraliakos X, Poddubnyy D, Schwarze I, Braun J, *et al.* SAT0391 Depressive symptoms are associated with higher disease activity and worse functional status in AxSpA: a cross-sectional analysis from rabbit-SpA. Ann Rheum Dis 2020;79(Suppl 1):1144–5.

191. Rondags A, Arends S, Wink F, Horvath B, Spoorenberg A. FRI0184 High prevalence of hidradenitis suppurativa, especially in female axial spondyloarthritis patients with high disease activity and poor quality of life. Ann Rheum Dis 2018;77(Suppl 2):633.

192. Rosenbaum JT, Pisenti L, Park Y, Howard R. Insight into the quality of life of patients with ankylosing spondylitis: real-world data from a US-based life impact survey [abstract]. Arthritis Rheumatol 2018;70(Suppl 9):1623.

193. Sagard J, Lindqvist E, Mogard E, Jöud A, Geijer M, Olofsson T, *et al.* Comorbidity burden in patients with non-radiographic axial spondyloarthritis at least as high as in ankylosing spondylitis [abstract]. Arthritis Rheumatol 2020;72(Suppl 10):1327.

194. Samreen S, ud din I, Gul H, Salim B, Parveen S, Nasim A. Impact of gender on clinical and radiological features amongst patients with axial spondyloarthropathy. Int J Rheum 2022:280.

195. Telugu S, Dekate P, Jayasridevi M, Dudam R. Assessment of quality of life in relation with disease activity indices, mobility and functionality in axial spondyloarthropathy – a cross-sectional study. Int J Rheum Dis 2021:280.

196. Ursin K, Lydersen S, Skomsvoll J, Wallenius M. THU0672 Impact of pregnancy on physical function and health-related quality of life in women with axial spondyloarthritis. Ann Rheum Dis 2018;77(Suppl 2):529–30.

197. van der Meer R, Arends S, Kruidhof S, Bos R, Bootsma H, Wink F, *et al.* FRI0210 Extra-articular manifestations are associated with worse quality of life and clinical outcome in patients with axial spondyloarthritis. Ann Rheum Dis 2018;77(Suppl 2):646.
